# GM-CSF and IL-21-armed oncolytic vaccinia virus significantly enhances anti-tumor activity and synergizes with anti-PD1 immunotherapy in pancreatic cancer

**DOI:** 10.3389/fimmu.2024.1506632

**Published:** 2025-01-03

**Authors:** Yujing Xuan, Wenyi Yan, Ruimin Wang, Xibin Wang, Yu Guo, Huilin Dun, Ziyan Huan, Lihua Xu, Ruxia Han, Xianlei Sun, Lingling Si, Nicholas R. Lemoine, Yaohe Wang, Pengju Wang

**Affiliations:** ^1^ Sino-British Research Centre for Molecular Oncology, National Centre for International Research in Cell and Gene Therapy, State Key Laboratory of Esophageal Cancer Prevention & Treatment, School of Basic Medical Sciences, Academy of Medical Sciences, Zhengzhou University, Zhengzhou, China; ^2^ Department of Pathology, Zhengzhou People’s Hospital, Fifth Clinical Medical College of Henan University of Chinese Medicine, Zhengzhou, China; ^3^ Centre for Biomarkers & Biotherapeutics, Barts Cancer Institute, Queen Mary University of London, London, United Kingdom

**Keywords:** oncolytic vaccinia virus, GM-CSF, IL-21, pancreatic cancer, anti-PD1

## Abstract

Pancreatic cancer is one of the most aggressive cancers and poses significant challenges to current therapies because of its complex immunosuppressive tumor microenvironment (TME). Oncolytic viruses armed with immunoregulatory molecules are promising strategies to overcome limited efficacy and target inaccessible and metastatic tumors. In this study, we constructed a tumor-selective vaccinia virus (VV) with deletions of the TK and A49 genes (VVLΔTKΔA49, VVL-DD) using CRISPR-Cas9-based homologous recombination. VVL-DD exhibited significant tumor selectivity *in vitro* and anti-tumor potency *in vivo* in a murine pancreatic cancer model. Then, VVL-DD was armed with an optimal combination of immunomodulatory molecules, granulocyte-macrophage colony-stimulating factor (GM-CSF) and interleukin-21 (IL-21), to produce VVL-GL21. VVL-GL21 induced significant tumor regression after intratumoral and systemic administration. Moreover, VVL-GL21 increased the infiltration of dendritic cells (DCs), macrophages, and T cells; induced DC maturation; increased the transition from M2 to M1 macrophages; improved the formation of immune memory; prevented tumor recurrence; and effectively bolstered the immune response against tumors in multiple key immune compartments. Interestingly, mice bearing-pancreatic cancer tumors treated with VVL-GL21 showed anti-tumor immunity against lung and colon cancer tumors. Importantly, treatment with VVL-GL21 enhanced the responsiveness of tumors to the immune checkpoint inhibitor anti-PD1. Taken together, VVL-GL21 remodels the suppressive TME and has powerful anti-tumor activities as monotherapy or in combination with anti-PD1 by intratumoral or systemic delivery for the treatment of pancreatic cancer. VVL-GL21 could be used as a therapeutic cancer vaccine.

## 1 Introduction

Approximately 20 million new cancer cases and 9.7 million deaths from cancer were reported worldwide in 2022, and pancreatic cancer (PaCa) was identified as the sixth leading cause of cancer-related mortality, with 511,000 new cases and 467,000 deaths (1). PaCa has the worst prognosis and survival rates among all cancers. Although immune checkpoint inhibitors (ICIs) achieve durable remission in various cancer types and have revolutionized cancer therapy, the objective response rate of advanced PaCa is approximately 10%. Chimeric antigen receptor (CAR)-engineered cell therapies for pancreatic cancer have not been approved for clinical use despite numerous clinical trials (2, 3). The effectiveness of these therapies against PaCa is limited by the hostile tumor microenvironment (TME) characterized by fibrosis, hypoxia, and immunosuppression, which includes a large number of M2-type macrophages and suppressive regulatory T cells (Tregs) (4).

Oncolytic viruses (OVs) are a promising and innovative cancer treatment strategy. By infecting cancer cells while sparing healthy tissues, oncolytic viruses can replicate, cause cell rupture, remodel the TME, facilitate T cell accumulation, and activate anti-tumor immune responses (5). Oncolytic Herpes simplex virus (HSV) encoding granulocyte-macrophage colony-stimulating factor (GM-CSF), T-VEC was approved in 2015 and has paved the way for the recognition of OV as validated therapeutics (6). A phase Ib clinical trial has shown that T-VEC may improve the efficacy of anti-programmed cell death receptor 1 (α-PD1) therapy by changing the TME, with a high response rate in patients with advanced melanoma (7).

Vaccinia virus (VV) is a double-stranded DNA virus that replicates in the cytoplasm. It has been used as a vaccine for smallpox and can be injected into tumor sites or intravenously to target metastatic tumors (8–10). Notably, the hypoxic environment in solid tumors does not affect VV replication (11). Despite the promising anti-tumor properties of VV, clinical efficacy needs to be improved by reducing viral virulence and optimizing the therapeutic molecules, in order to maximize benefits and minimize adverse effects (12). JX-594 (Pexa-Vec; Jennerex Inc.), a thymidine kinase (TK, J2R) gene-inactivated Wyeth strain VV expressing GM-CSF, selectively targets cancer cells and has shown promising safety and efficacy in phase II trials using intratumoral or intravenous delivery to treat soft tissue sarcomas (13, 14). GM-CSF induces monocyte differentiation into dendritic cells (DCs) and macrophages, enhances DC survival, induces DC maturation, and promotes cytokine and chemokine production. However, the PHOCUS study, a randomized phase III clinical trial for sequential treatment with JX-594 and sorafenib did not show increased clinical benefits in advanced hepatocellular carcinoma, with the results for the combined treatment being worse compared to sorafenib administered alone (15). Clinical trials of JX-594 and T-VEC have demonstrated that oncolytic viruses containing GM-CSF are safe, but their anti-tumor efficacy needs to be improved.

We previously reported that the Lister strain VV (VVL) with TK gene deletion (VVLΔTK) is a tractable platform for the development of a new generation of oncolytic VVs (16). VV protein A49, a small intracellular protein with a B cell lymphoma (Bcl)-2-like fold, promotes virus virulence and inhibits NF-κB activation (17). VVs engineered to lack A49 exhibit lower virulence than wild-type viruses (17). Therefore, deletion of TKs and A49 may enhance the immunogenicity and reduce the virulence of VVL. VVL-expressing interleukin-21 (IL-21) exhibits good safety and anti-tumor efficacy against various types of tumors (18). IL-21 is crucial to promote the proliferation and cytotoxicity of T cells and natural killer T (NKT) cells, memory T cell differentiation, macrophage transition from M2 to M1, and angiogenesis inhibition *in vivo (* 19). Importantly, high doses of IL-21 doses are not associated with adverse effects. However, clinical trials have shown limited effectiveness of stand-alone anti-tumor therapy (20). The combination of GM-CSF and IL-21 may activate both innate and adaptive immunity, potentially reversing the immunosuppressive TME and enhancing anti-tumor immunity.

Here, we report a re-engineering of VVL with deletion of the TK and A49 genes (VVLΔTKΔA49, double deletion, VVL-DD) that showed high tumor selectivity and promising anti-tumor potency. We armed VVL-DD with an optimal combination of GM-CSF and IL-21 to create VVLΔTK-GM-CSFΔA49-IL-21 (hereafter, VVL-GL21), and investigated its function in remodeling TME as well as its anti-tumor efficacy when administered either alone or in combination with α-PD1 via intratumoral and intravenous delivery in the PaCa mouse model.

## 2 Materials and methods

### 2.1 Cell lines

CV1 (African monkey kidney) cells were obtained from the American Type Culture Collection (ATCC, VA, USA). DT6606 and DT4994 cells are pancreatic ductal adenocarcinoma (PDAC) cell lines derived from LSL-KrasG12D; Pdx-1-Cre mice that had developed PDAC. TB11381 cells are a PDAC cell line derived from Pdx-1-Cre/KrasG12D/p53R172H mice that had developed PDAC. DT6606, DT4994, and TB11381 cells were gifted by Professor David Tuveson (Cambridge Research Institute). MC38 (mouse colon carcinoma cells), CMT64 (mouse lung carcinoma cells), TC-1 (mouse lung epithelial cells), MRC-5 (human lung fibroblasts), HeLa (human cervical cancer cell), HepG2 (hepatocarcinoma cells), EC1 (human esophageal cancer cells), SKOV3 (human ovarian cancer cells), and SUIT-2 (human pancreatic cancer cells) cells were obtained from the Cell Resource Center, Peking Union Medical College (part of the National Science and Technology Infrastructure, the National Biomedical Cell-Line Resource, NSTI-BMCR. http://cellresource.cn. CV1, DT6606, TB11381, DT4994, MRC-5, HeLa, HepG2, SKOV3, EC1, and CMT-64 cells were cultured in Dulbecco’s modified Eagle medium (DMEM; Cat# 11965092, Gibco-Thermo Fisher Scientific, USA) supplemented with 10% fetal bovine serum (FBS; Cat#16000044, Gibco), 1% streptomycin, and 1% penicillin. SUIT-2, MC38, and TC-1 cells were cultured in RPMI-1640 medium (Sigma-Aldrich) supplemented with 10% FBS, 1% streptomycin, and 1% penicillin. All cells were incubated at 37°C with 5% CO_2_ atmosphere.

### 2.2 Recombinant VV construction, purification, and expansion

To generate recombinant oncolytic VVs, a Lister strain was used as the parental virus for homologous recombination using the CRISPR/Cas9 system (21). The specific alterations introduced in the virus involved the removal of the TK gene and its replacement with the GM-CSF gene, while simultaneously deleting the A49 gene and replacing it with the either the IL-21 or the red fluorescent protein (RFP) genes. The shuttle vectors pTK and pA49 were synthesized by GENEWIZ (Azenta Life Sciences, Suzhou, China). The pTK vector containing RFP flanked by LoxP sites was used, as previously described (22). The pA49 vector containing RFP flanked by FRT sites was modified similarly to the N1L region, as previously described (22). Murine GM-CSF and IL-21 genes were synthesized by GENEWIZ with Sal I and Nhe I sites in the pUC57 vectors. GM-CSF and IL-21 were released from pUC57 using Sal I and Nhe I restriction enzymes and cloned into the Sal I and Nhe I sites of the pTK and pA49 shuttle vectors, respectively. Guide RNA (gRNA) sequences for the TK and A49 regions of VV were selected as previously described (23). gRNA oligos with extra ends were synthesized (GENEWIZ, China) and cloned into the gRNA cloning vector PB-gRNA-Bsa1 (24). Viral construction and production were performed in CV1 cells as previously described (25).

### 2.3 Vaccinia virus replication and cell cytotoxicity assays

Tumor cells were infected with viruses at a multiplicity of infection (MOI) of 1 PFU/cell. The cells and supernatants were collected at 24-, 48-, and 72-h post-infection. The median tissue culture infective dose (TCID50) for each sample was calculated by detecting the cytopathic effect seven days after infection, as described previously for indicator CV1 cells.

The cytotoxicity of the viruses in each cell line was assessed in triplicate 6 d after infection using an MTS non-radioactive cell proliferation assay kit (Promega) according to the manufacturer’s instructions.

### 2.4 ELISA

Tumor cells were seeded in triplicate and infected with viruses 16 h later at an MOI of 1 PFU/cell. Supernatants were collected at 24-, 48-, and 72-h post-infection and cytokine levels were measured using ELISA. GM-CSF (Invitrogen) and IL-21 (Invitrogen) levels were detected using ELISA in accordance with the manufacturer’s instructions.

Six-week-old male C57BL/6 mice were subcutaneously injected with 2 × 10^6^ DT6606 cells. VVL-GL21 or phosphate-buffered saline (PBS) was injected intratumorally on day 7. Blood samples and subcutaneous tumors were collected at 24 h post-injection. The serum was extracted by centrifugation at 3000 rpm for 20 min at 4°C from blood samples laid 2 h at room temperature. Cytokine levels in the tumor homogenates and serum samples were evaluated using ELISA, in accordance with the manufacturer’s instructions.

### 2.5 *In vivo* studies

All animals were obtained from Vital River Corp. (Beijing, China). All experiments on mice were approved by the Animal Welfare and Research Ethics Committee of Zhengzhou (Zhengzhou, China) for the 2019YFC1316101 project to PW (ID: V3A02022000001). Tumor volume (V = π × length × width × width/6) was measured until the tumor volume reached 2000 mm^3^ or the tumor ulcerated, at which point the animals were euthanized. Mice were randomly grouped based on tumor sizes. Tumor growth curves were stopped after the first animal in each group died or was sacrificed. Animal survival was monitored until the experimental endpoint.

### 2.6 Treatment of subcutaneous PaCa or colon cancer

Six-week-old male C57BL/6 mice were subcutaneously implanted with 2 × 10^6^ DT6606 cells. Seven days later, treatment was initiated, when the average tumor volume was about 100-120 mm^3^. Ten days later, when the average tumor volume was about 160 mm^3^, treatment was initiated in advanced cancers. Six or seven mice per group were injected intratumorally or intravenously with different viruses or PBS on days 0, 2 and 4. When virotherapy was combined with ICI, α-PD1 (clone 29F.1A12, Bio X Cell) was administered at a dose of 200 μg/injection in 100 μL PBS via intraperitoneal injection on days 4, 7, 11, and 14 (n=7).

Six-week-old female C57BL/6 mice were subcutaneously implanted with 1.5 × 10^6^ TB11381 cells. Seven days later, when tumor xenograft volume reached approximately 160 mm^3^, PBS or other viruses were intratumorally injected on days 0, 2, and 4 (n=7).

Six-week-old female C57BL/6 mice were subcutaneously implanted with 5 × 10^5^ murine colorectal cancer MC38 cells. Seven days later, when the tumor xenograft volume reached approximately 100 mm^3^, PBS or other viruses were intratumorally injected on days 0, 2, and 4 (n=7).

### 2.7 Immune cell depletion

When the subcutaneous DT6606 xenograft volume reached approximately 122 mm^3^, 250 μg of anti-CD8 IgG (clone TIB210), anti-CD4 IgG (clone GK1.5), anti-NK IgG (clone PK136), control IgG, or 200 μL of clodronate liposomes (Yeasen) and control liposomes (Yeasen) were injected intraperitoneally to deplete CD8+ T cells, CD4+ T cells, NK cells, or macrophages. Liposomes were injected once a week, and anti-body injections were continued twice a week during the experiment. Depletion was verified by fluorescence-activated cell sorting (FACS) assessment of blood or spleen cell populations 24 h after administration. Intratumoral injections containing different viruses or PBS were administered three times every other day starting 24 h after the first depletion. Tumor size was measured twice weekly using digital calipers.

### 2.8 Immunohistochemistry

DT6606 subcutaneous tumors were collected and immediately fixed in a 4% paraformaldehyde solution for 48 h. Samples were embedded in paraffin, followed by immunohistochemical (IHC) staining to detect CD8 expression (Servicebio, GB15068-50). In brief, 3-μm paraffin sections were prepared, blocked with 3% bovine serum albumin, incubated with the 300× diluted anti-body, washed, incubated with anti-rabbit anti-body conjugated with HRP, and stained with DAB chromogen solution and followed by hematoxylin counterstain. Imaging of the slides was performed with a KONFOONG Digital Pathological Slice Scanner (KF-PRO-005, Konfoong bioinformation tech CO., LTD). Images were analyzed using the Indica lab HALO pathological section analysis platform.

### 2.9 FACS analysis

The tumors, spleens, and draining lymph nodes were extracted from the mice. Tumors were homogenized and incubated in collagenase IV (1 mg/mL, Sigma), collagenase I (1 mg/mL, Sigma) in RPMI-1640, at 37°C for 40 min on a shaker. Homogenates were filtered through 70-μm cell strainers. Spleens and draining lymph nodes were smashed and pushed through a 70-μm cell strainer to create a single cell suspension. Splenocytes were centrifuged, and the pellet was incubated for 10 min in 5 ml red blood cell lysis buffer (Beyotime). Tumor cells, splenocytes, and lymph node cells (1 × 10^6^) were washed with PBS twice then stained with Zombie NIR™ Fixable Viability Kit (Biolegend, 423105), TruStain FcXTM Antibody (Biolegend, 101320), PerCP/Cyanine5.5 anti-mouse CD45 (Biolegend, 103132), FITC anti-mouse CD3 (Biolegend, 100204), Brilliant Violet 510™ anti-mouse CD4 (Biolegend, 100559), Brilliant Violet 650™ anti-mouse CD8a (Biolegend, 100742), Brilliant Violet 421™ anti-mouse NK-1.1 (Biolegend, 108741), PE/Dazzle™ 594 anti-mouse/human CD44 (Biolegend, 103055), Brilliant Violet 785™ anti-mouse CD62L (Biolegend, 104440), PE/Cyanine7 anti-mouse CD122 (Biolegend, 123215), FITC anti-mouse/human CD11b (Biolegend, 101206), Brilliant Violet 785™ anti-mouse CD86 (Biolegend, 105043), PE/Cyanine7 anti-mouse F4/80 (Biolegend, 123114), Brilliant Violet 421™ anti-mouse CD11c (Biolegend, 117343), and APC anti-mouse I-A/I-E (Biolegend, 107614), PE/Cyanine7 anti-mouse CD40 (Biolegend, 124621), APC anti-mouse PD-1 (Biolegend, 109111), Brilliant Violet 421™ anti-mouse Tim3 (Biolegend, 119723), Alexa Fluor 488 conjugate TCF1/TCF7 Rabbit mAb (Cell signaling technology, 6444S). Cells were acquired on the BD FACS scanner and data were analyzed using FlowJo software (Tree Star Inc.).

### 2.10 IFN-γ release assay of spleen cells

Harvested spleens were crushed and flushed through 70-μm BD Falcon cell strainers. Red blood cells were lysed using RBC lysis buffer (Beyotime) and washed with PBS. Splenocytes were resuspended in complete T cell medium (TCM) containing RPMI-1640, 10% FBS, 1% streptomycin/penicillin, 1% sodium pyruvate, and 1% non-essential amino acids (Gibco). Then, 5 × 10^5^ splenocytes (100 μL) were added into each well of a 96-well plate and co-cultured with 10 μg chicken ovalbumin (OVA) peptide (SIINFEKL, 100 μL), 10 μg B8R peptide per well (TSYKFESV, 100 μL), 5 × 10^4^ Mitomytin C-DT6606 cells, Mitomytin C-CMT64 cells, or Mitomytin C-MC38 cells (100 μL). After 72 h, the 96-well plate was centrifuged at 1,600 rpm for 5 min, and the supernatant was collected to detect the concentration of IFN-γ using ELISA assay according to the manufacturers’ instructions (Invitrogen; 88-7314-88; Thermo Fisher Scientific). Data were analyzed as previously reported.

### 2.11 Detection and quantification of PD-L1 expression via FACS and western blot

A total of 2 × 10^5^ DT6606 cells were seeded into six-well plates containing the appropriate units/ml (U/mL) of IFN-γ. After 60 h, the cells were collected and stained with anti-mouse PD-L1 (124307, Biolegend). Cells were acquired on the BD FACS scanner, and data were analyzed using FlowJo software (Tree Star Inc.).

Six-week-old male C57BL/6 mice were subcutaneously injected with 2 × 10^6^ DT6606 cells. When the tumor xenografts reached approximately 150 mm^3^ in volume, VVL-GL21 or PBS was injected intratumorally on days 0 and 2, respectively. As mentioned above, subcutaneous tumors were collected and digested into single-cell suspensions on day 4. Cells were processed for FACS analysis as previously described. Total protein from tumor tissues was extracted to detect PD-L1 expression via western blotting using an anti-mouse PD-L1 antibody (clone 10F.9G2, Bio X Cell). GADPH (glyceraldehyde-3-phosphate dehydrogenase) was used as the endogenous control. The conditions were evaluated in triplicate. The density of the protein bands was quantified using ImageJ software (NIH, 1.53a) software.

### 2.12 Rechallenge of tumor-free animals

The C57BL/6 mice that underwent complete subcutaneous tumor regression following VVL-GL21(intratumoral) plus IgG (n = 4), VVL-GL21(intratumoral) plus α-PD1 (n = 7), or VVL-GL21(intravenous*)* plus α-PD1 (n = 6) treatment were rechallenged with 4 × 10^6^ DT6606 (twice the number of cells compared to the primary tumor cell inoculation), 5 × 10^6^ CMT64, or 5 × 10^5^ MC38 cells after primary tumors had been cleared for 42 d or 50 d. Tumor volumes were measured twice per week.

### 2.13 Statistical analysis

GraphPad Prism 9 was used for comparative statistical analysis. The results are presented as mean ± SEM. Comparison between the groups was performed using Student’s t-test, one-way analysis of variance (ANOVA), or two-way ANOVA. Survival data were analyzed using Kaplan–Meier plots and log-rank analysis to determine the significance of differences between specific treatment pairs (10). A p-value < 0.05 was considered significant.

## 3 Results

### 3.1 VVL-DD is an effective anti-tumor therapy in murine PaCa models

**Figure 1:**
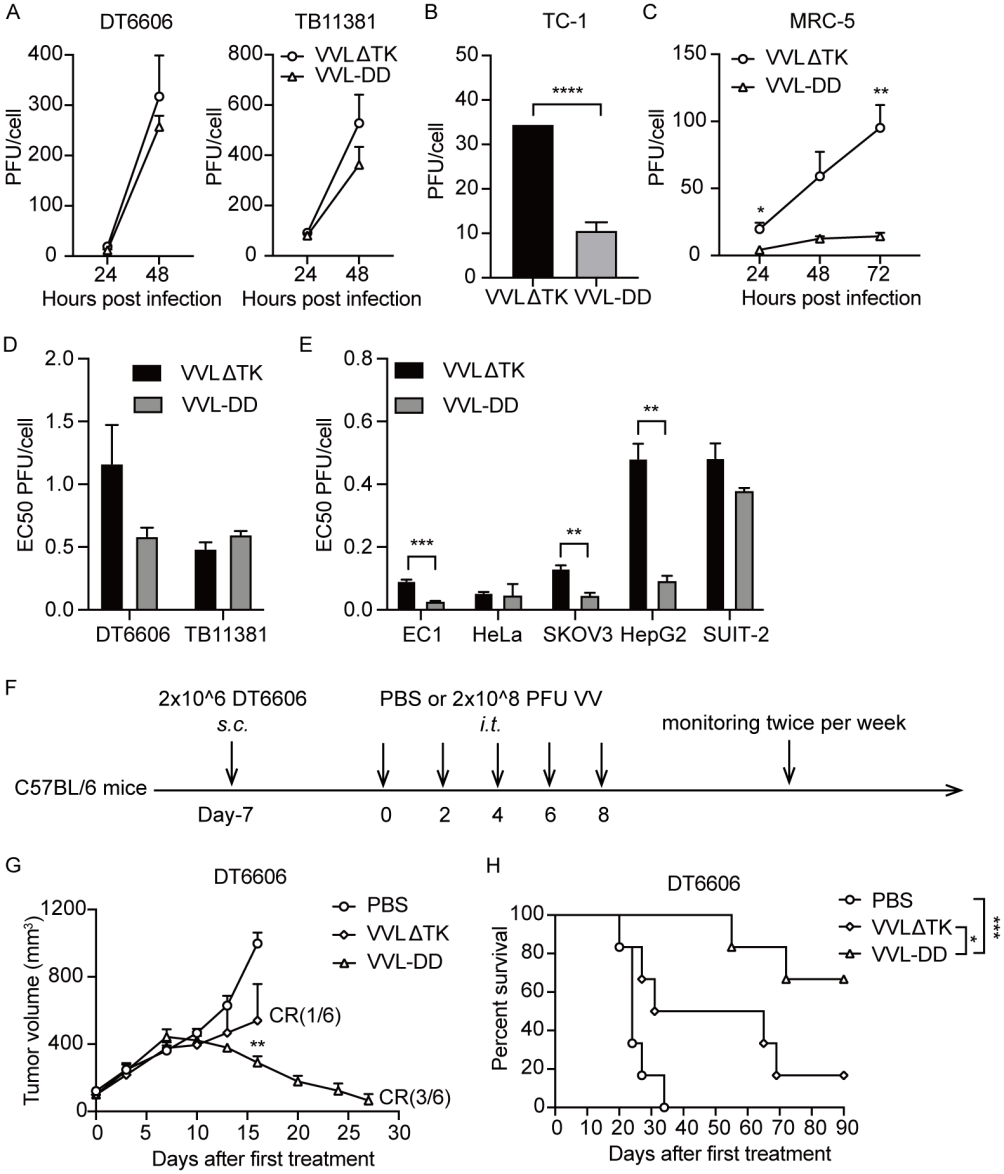
Deletion of the A49 gene of VVLΔTK improves tumor selectivity and anti-tumor efficacy of VV. Virus replication was determined in murine DT6606 and TB11381 **(A)**, mouse lung epithelial cells TC-1 **(B)**, and human lung fibroblasts MRC-5 **(C)** cells over 24–72 h Virus production was determined via titration of a mixture of the supernatant and whole cell lysates (n = 3). **(D, E)** The EC50 value (dose required to kill 50% of the cells) of VVLΔTK and VVL-DD was compared using MTS assays in murine and human cell lines (n = 3). **(F)** Schematic for the *in vivo* treatment protocol. Wild-type mice were subcutaneously engrafted with DT6606 cells. Seven days later (average tumor size 100 mm^3^), mice were treated with intratumoral injection of VVLΔTK or VVL-DD (2 × 10^8^ PFU/injection) on days 0, 2, 4, 6, and 8 (n = 6). **(G)** Tumor size was monitored twice weekly. **(H)** Survival curve of DT6606 tumors. Data are presented as the mean ± SEM. The data in **(A–E)** were analyzed using an unpaired Student’s t-test. The data in **(G)** were analyzed using a one-way ANOVA with Tukey’s multiple comparison post-test. The data in **(H)** were analyzed using Kaplan–Meier survival analysis with log rank (Mantel-Cox) tests. *p < 0.05, **p < 0.01, ***p < 0.001, ****p < 0.0001.

Replication of VVL-DD was not significantly attenuated in murine PaCa cell lines, but significantly attenuated in the mouse lung epithelial cells TC-1 and in the human lung fibroblasts MRC-5 (**Figures 1A–C**). Deletion of A49 did not reduce cytotoxicity of VVL-DD compared to VVLΔTK in any of the murine or human tumor cell lines (**Figures 1D, E**). *In vivo*, VVL-DD exerted significantly greater control over the growth of murine pancreatic DT6606 subcutaneous tumors compared to VVLΔTK, with improved survival and tumor free rate in the model (**Figures 1F–H**).

### 3.2 VVL-GL21 replicates, causes cell death, and produces GM-CSF and IL-21 in murine PaCa cell lines

**Figure 2:**
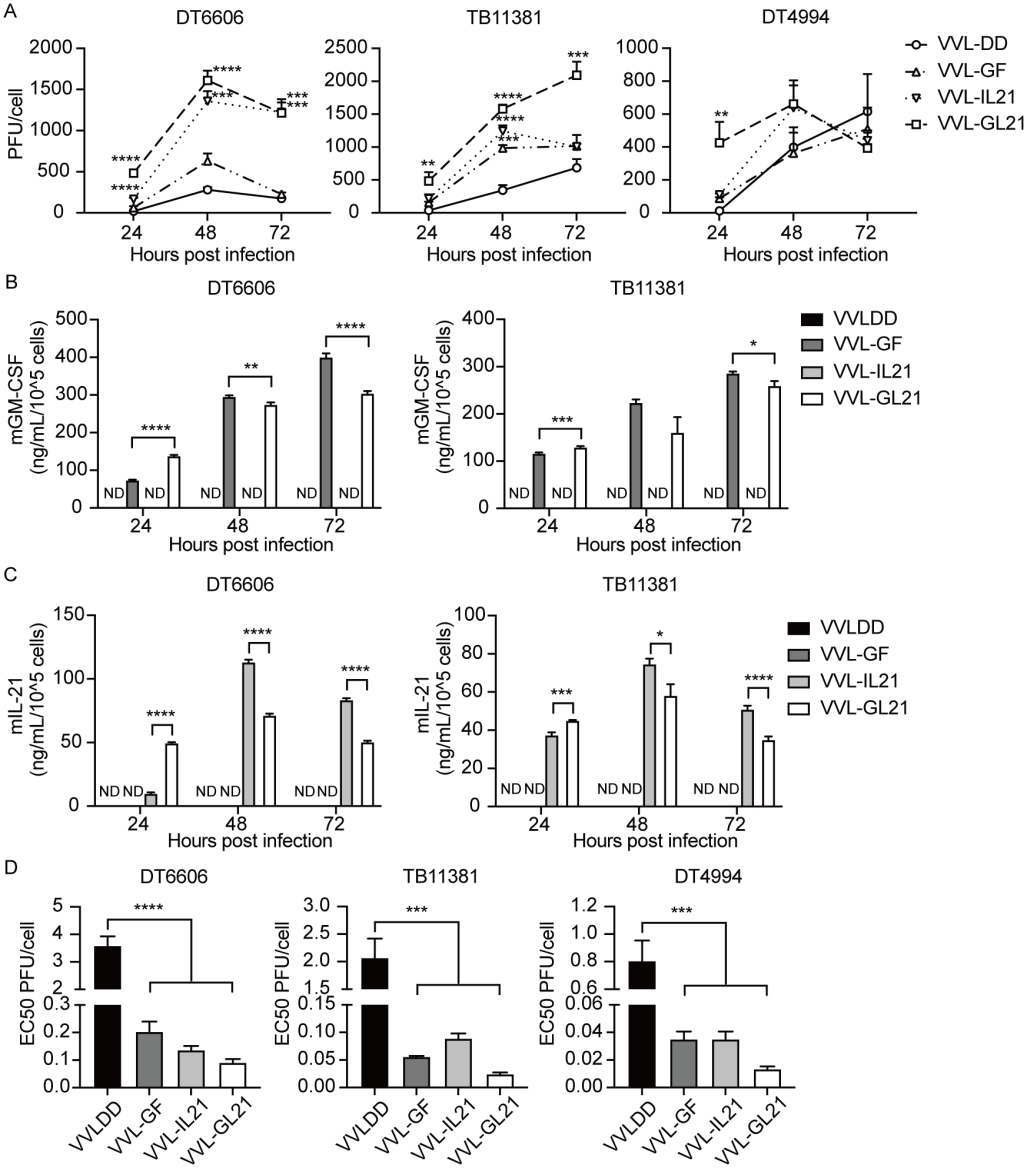
VVL-GL21 remains cytotoxic and replication competent in murine PaCa cell lines. **(A)** Virus replication was determined in DT6606, TB11381, and DT4994 cells over 24–72 h (n = 3). Significance is shown in relation to VVL-DD group. **(B, C)** Murine GM-CSF and IL-21 expression was detected in the supernatant from the indicated cell cultures at 24, 48, and 72 h post-infection with 1PFU/cell VVL-DD, VVL-GF, VVL-IL21 or VVL-GL21 virus using ELISA (n = 3). **(D)** The EC50 value (dose required to kill 50% of cells) of VVL-DD, VVL-GF, VVL-IL21, and VVL-GL21 was compared using MTS assays in DT6606, TB11381, and DT4994 cell lines (n = 3). Data are presented as the mean ± SEM. The data in **(A, D)** were analyzed using one-way ANOVA and Tukey’s multiple comparison post-test. The data in **(B, C)** were analyzed using an unpaired Student’s t-test. *p < 0.05, **p < 0.01, ***p < 0.001, ****p < 0.0001. ND, not detected.

To further enhance anti-tumor efficacy, VVL-GL21 was created using CRISPR/Cas9-based homologous recombination to insert the GM-CSF or IL-21 gene into the TK or A49 region of the VVL genome (22, 26). VVLΔTK-GM-CSFΔA49 and VVLΔTKΔA49-IL-21 (referred to hereafter as VVL-GF and VVL-IL21, respectively) made specific alterations that detailed the construction of mutant viruses (**Supplementary Figure S1**). The 50% tissue culture infectious dose (TCID50) assay of viral titers at each time point indicated that all viruses replicated effectively in DT6606, DT4994, and TB11381 cells (**Figure 2A**). GM-CSF and IL-21 expression was confirmed in the cell supernatant using ELISA (**Figures 2B, C**). All viruses exhibited low EC50 values, indicating high cytotoxicity against the tumor cell lines (**Figure 2D**). A significant difference in cytotoxicity was observed between VVL-DD and GM-CSF- and/or IL-21-armed viruses, indicating that the addition of GM-CSF and/or IL-21 affected the viral cytotoxic effect *in vitro* (**Figure 2D**).

### 3.3 VVL-GL21 improves anti-tumor efficacy in murine subcutaneous tumors

**Figure 3:**
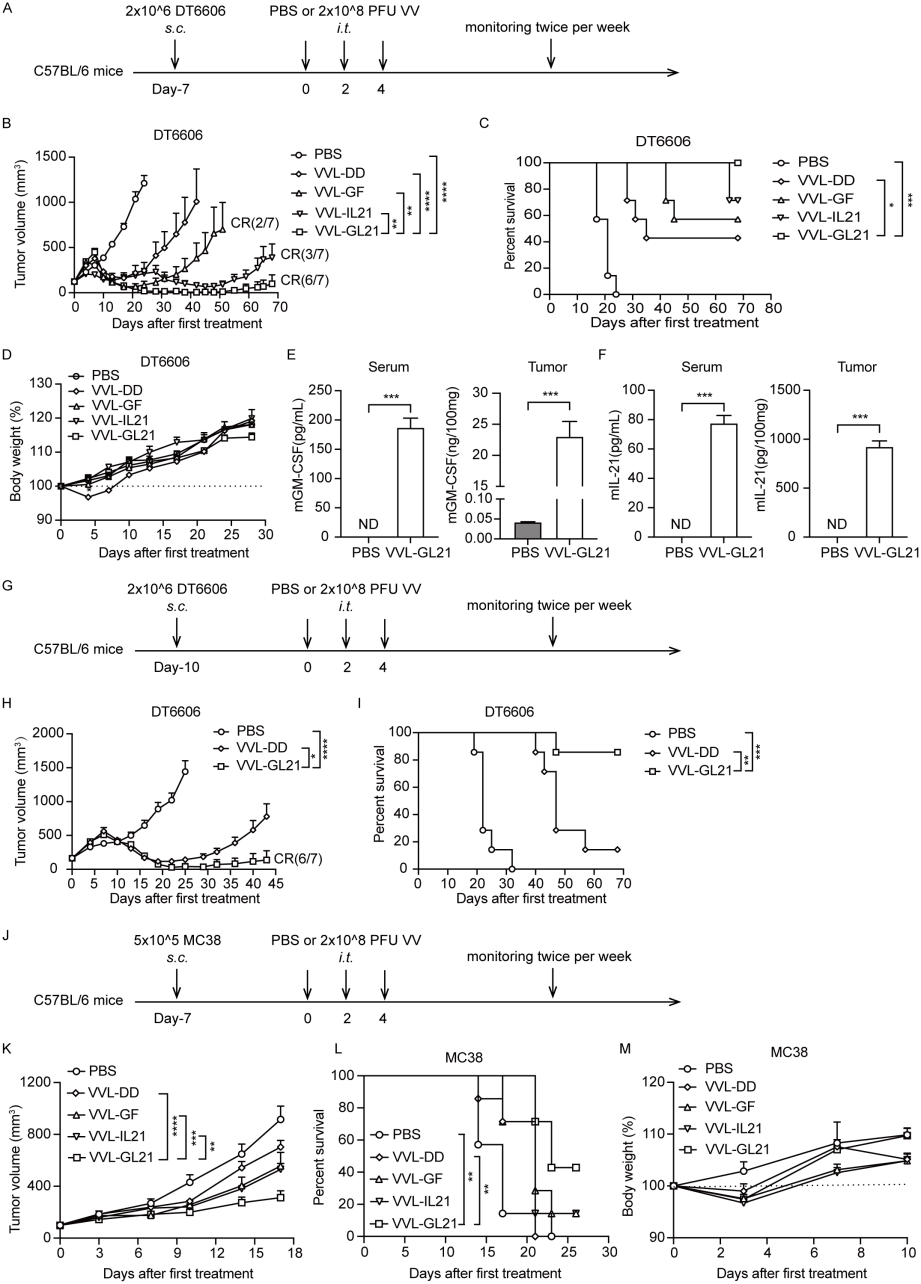
VVL-GL21 improves *in vivo* anti-tumor efficacy in murine pancreatic cancer and colon cancer models. **(A)** Schematic for the *in vivo* treatment protocol. Wild-type mice were subcutaneously engrafted with 2 × 10^6^ DT6606 cells. Seven days later (average tumor size 120 mm^3^), mice were treated with intratumoral injection of VVL-DD, VVL-GF, VVL-IL21, or VVL-GL21 (2 × 10^8^ PFU/injection) on days 0, 2, and 4 (n = 7). **(B)** Tumor growth curve. **(C)** Survival curve. **(D)** Body weight measurements. **(E, F)** Tumors were homogenized and serum was collected at 24 h after the treatment. GM-CSF and IL-21 expression was detected using ELISA (n = 3). **(G)** Schematic for the *in vivo* treatment protocol. DT6606 tumors were established subcutaneously and once palpable (160 mm^3^), were treated with intratumoral injection of VVL-DD or VVL-GL21 (2 × 10^8^ PFU/injection) on days 0, 2, and 4 (n = 7). **(H)** Tumor growth curve. **(I)** Survival curve. **(J)** Schematic for the *in vivo* treatment protocol. MC38 tumors were established subcutaneously and once palpable (100 mm^3^), were treated with intratumoral injection of VVL-DD, VVL-GF, VVL-IL21, or VVL-GL21 (2 × 10^8^ PFU/injection) on days 0, 2, and 4 (n = 7). **(K)** Tumor growth curve. **(L)** Survival curve. **(M)** Body weight measurement. Data are presented as the mean ± SEM. The data in **(B, E, F, H)** were analyzed using an unpaired Student’s t-test. The data in **(D, K, M)** were analyzed using two-way ANOVA with Tukey’s multiple comparison post-test. The data in **(C, I, L)** were analyzed using Kaplan–Meier survival analysis with log rank (Mantel-Cox) tests. *p < 0.05, **p < 0.01, ***p < 0.001, ****p < 0.0001. ND, not detected.

Subcutaneous DT6606 tumors were established in immunocompetent mice and treated with three intratumoral injections of PBS or viruses (2 × 10^8^ PFU/injection) on days 0, 2, and 4 (**Figure 3A**). VVL-GF and VVL-IL21 delayed tumor growth and cured 2/7 and 3/7 mice, respectively. The therapeutic virus (VVL-GL21) was significantly more effective than either of these treatments, with complete tumor clearance and long-term survival observed in 6/7 mice treated (**Figures 3B, C**). Sera and tumors were collected at 24 h after the treatment. GM-CSF and IL-21 expression was detected in homogenized tumors and sera by ELISA. The expression of GM-CSF and IL-21 was stable in the tumors (**Figures 3E, F**). VVL-GL21 showed significantly superior anti-tumor efficacy against tumors with a high burden (160 mm^3^) cured 6/7 mice (**Figures 3G–I**). Furthermore, the *in vivo* anti-tumor efficacy of VVL-GL21 was confirmed in a murine PaCa TB11381 model (**Supplementary Figure S2**).

We also evaluated the efficacy of VVL-GL21 in a murine colorectal cancer MC38 model (**Figure 3J**). VVL-GL21 significantly inhibited tumor growth and improved survival (**Figures 3K, L**). VVL-GL21 did not significantly affect the weight of mice (**Figures 3D, M**, **Supplementary Figure S2D**). Meanwhile, no pathological changes were observed in the lungs, liver, kidneys, and heart upon treatment with VVL-GL21 (**Supplementary Figure S4E**).

### 3.4 VVL-GL21 modulates the TME, draining lymph nodes, and splenic lymphocyte subsets

**Figure 4:**
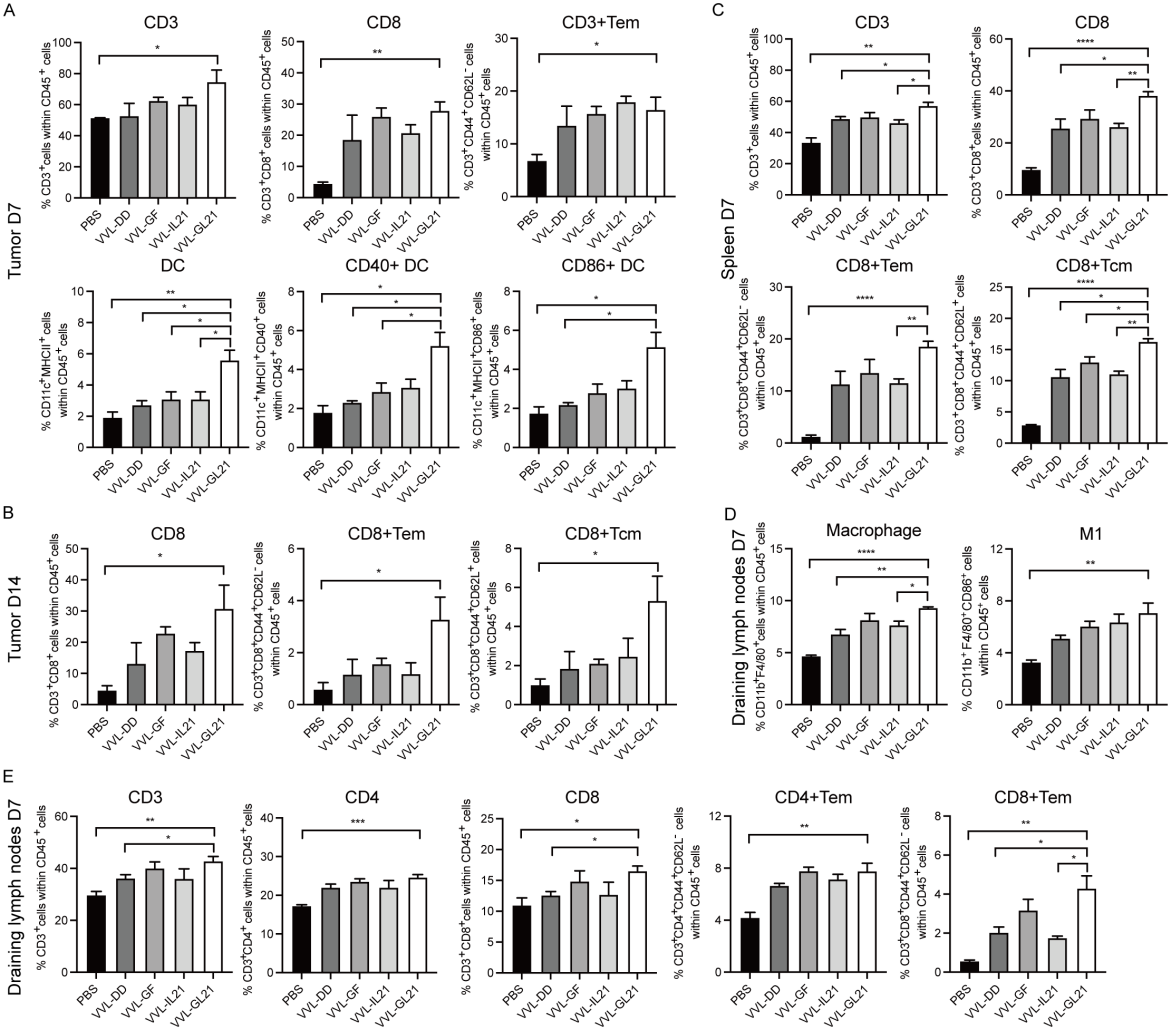
VVL-GL21 induces robust anti-tumor immune responses. Wild-type mice were subcutaneously engrafted with 2 × 10^6^ DT6606 cells. Seven days later, mice were treated with 2 × 10^8^ PFU of VVL-DD, VVL-GF, VVL-IL21, or VVL-GL21. **(A–E)** Tumors, spleens, and draining lymph nodes were collected from mice on day 7 and 14 post-treatment. FACS was performed to analyze immune cell composition using single-cell suspensions prepared from these tissues (n = 3). Data are presented as the mean ± SEM. The data in **(A–E)** were analyzed using an unpaired Student’s t-test. *p < 0.05, **p < 0.01, ***p < 0.001, ****p < 0.0001.

To elucidate the therapeutic effects of VVL-GL21, we detected T cells, macrophages, and DC populations infiltrating DT6606 subcutaneous tumors. FACS analysis showed that VVL-GL21 increased the number of DCs and activated DCs within the tumor on day 7 after treatment (**Figure 4A**). CD8+ T cell infiltration into tumors increased after VVL-GL21 treatment from 7^th^ day to 14^th^ day, as detected by FACS and immunohistochemistry (IHC) (**Figures 4A, B**, **Supplementary Figures S4A, B**). VVL-GL21 treatment increased the number of effector memory T cell (Tem) subsets on day 7 and increased the CD8+ Tem and CD8+ central memory (Tcm) subset infiltration on day 14 (**Figures 4A, B**).

Next, changes in T cell subsets were examined following intratumoral virus injection in the spleen. On day 7, total CD3+, CD8+, CD8+ Tem, and CD8+ Tcm cells in the spleens of mice treated with the viruses were significantly increased compared to those of mice treated with PBS (**Figure 4C**). An increase in splenic CD4+ Tem, CD8+ Tem, and NKT cells was observed on day 14 after VVL-GL21 treatment compared with the control group (**Supplementary Figure S4C**).

We also assessed the T cell and macrophage populations in draining lymph nodes on day 7 after virus treatment. The total number of CD3+, CD4+, CD4+ Tem, CD8+, and CD8+ Tem cells, macrophages, and M1 macrophages from draining lymph nodes in mice treated with VVL-GL21 was significantly higher than that in mice treated with PBS (**Figures 4D, E**). The numbers of CD4+ Tem cells, CD8+ Tem cells, and M1 macrophages remained high on day 14 (**Supplementary Figure S4D**).

We also measured T cells infiltrating MC38 subcutaneous tumors. FACS analysis showed that VVL-GL21 increased the number of CD4+ and CD8+ T cells within the tumor after treatment (**Supplementary Figure S4F**).

These findings suggest that the inhibitory effect of VVL-GL21 on tumor growth may be attributed to its ability to reverse the suppressive characteristics of the TME.

### 3.5 VVL-GL21 activates the anti-viral and anti-tumor immunity of splenic T cells

**Figure 5:**
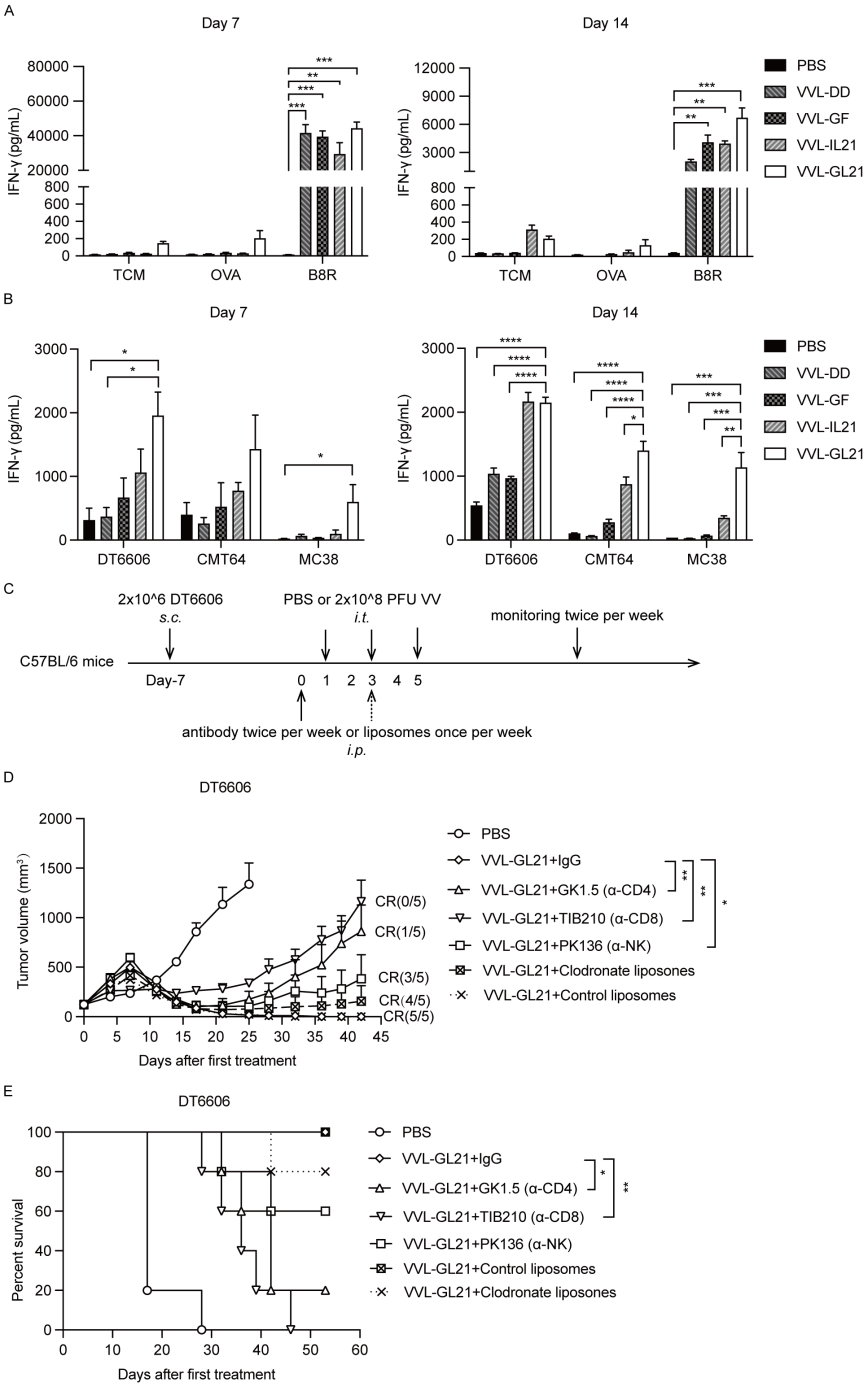
Immune subsets affect the anti-tumor efficacy of VVL-GL21. **(A, B)** Wild-type mice were subcutaneously engrafted with 2 × 10^6^ DT6606 cells. Seven days later, mice were treated with 2 × 10^8^ PFU of VVL-DD, VVL-GF, VVL-IL21, or VVL-GL21. Splenocytes were harvested on days 7 and 14 post-treatment and restimulated with the indicated agents. 72 h after re-stimulation, an ELISA for IFN-γ was performed (n = 3). **(C)** Schematic representation of the *in vivo* treatment protocol. DT6606 tumors were established subcutaneously and once palpable (120 mm^3^), were treated with intraperitoneal injections of anti-bodies twice a week or liposomes once a week for the duration of the experiment from day 0. Tumors were then treated with an intratumoral injection of VVL-GL21 (2 × 10^8^ PFU/injection) on days 1, 3, and 5 (n = 5). **(D)** Tumor growth curves. **(E)** Survival curves. Data are presented as the mean ± SEM. The data in **(A, B)** were analyzed using one-way ANOVA and Tukey’s multiple comparison test. The data in **(D)** were analyzed using an unpaired Student’s t-test. The data in **(E)** were analyzed using Kaplan–Meier survival analysis with log-rank (Mantel-Cox) tests. *p < 0.05, **p < 0.01, ***p < 0.001, ****p < 0.0001.

To determine the activity of VVL-GL21, IFN-γ production of splenocytes was evaluated *in vitro*. T cell medium, VV peptide B8R, chicken ovalbumin (OVA), mouse PaCa DT6606, mouse lung cancer CMT64, and mouse colon cancer MC38 cells were used to stimulate splenocytes collected on days 7 and 14 after treatment ex vivo, and IFN-γ production was assessed 72 h later. IFN-γ secretion significantly increased after stimulation with peptide B8R, suggesting an anti-viral response following treatment (**Figure 5A**). Stimulation with DT6606 cells induced IFN-γ in treatment groups, with the VVL-GL21 group showing the most significant increase (**Figure 5B**). The VVL-GL21 group exhibited high levels of IFN-γ from day 7 to day 14, whereas the VVL-IL21 group showed similar levels of IFN-γ on day 14 (**Figure 5B**). These results indicate that treatment with VVL-GL21 activates an earlier and stronger anti-tumor response than that in other groups. Higher levels of IFN-γ were detected after stimulation with CMT64 and MC38 cells in the splenic T cells of mice treated with VVL-GL21 than in other groups, indicating that VVL-GL21 generates anti-tumor immunity against other types of tumors and that cross-antigens may exist in DT6606, CMT64, and MC38 cells (**Figure 5B**).

### 3.6 VVL-GL21 efficacy depends on CD8+ T, CD4+ T, and natural killer cells

To assess the individual contribution of different immune cells to the anti-tumor effect of VVL-GL21, macrophages, CD8+ T cells, CD4+ T cells, and natural killer (NK) cells were depleted, and subset depletion was confirmed using FACS (**Supplementary Figure S5**). Macrophage depletion did not affect the efficacy of VVL-GL21, whereas NK cell depletion modestly affected treatment efficacy (**Figures 5C, D**). However, depletion of CD4+ T and CD8+ T cells negatively affected the ability of VVL-GL21 to control tumor growth and survival (**Figures 5D, E**), demonstrating that the anti-tumor effects of VVL-GL21 in this model are primarily mediated by CD8+ T, CD4+ T, and NK cells.

### 3.7 VVL-GL21 enhances the therapeutic effect of the checkpoint inhibitor α-PD1

**Figure 6:**
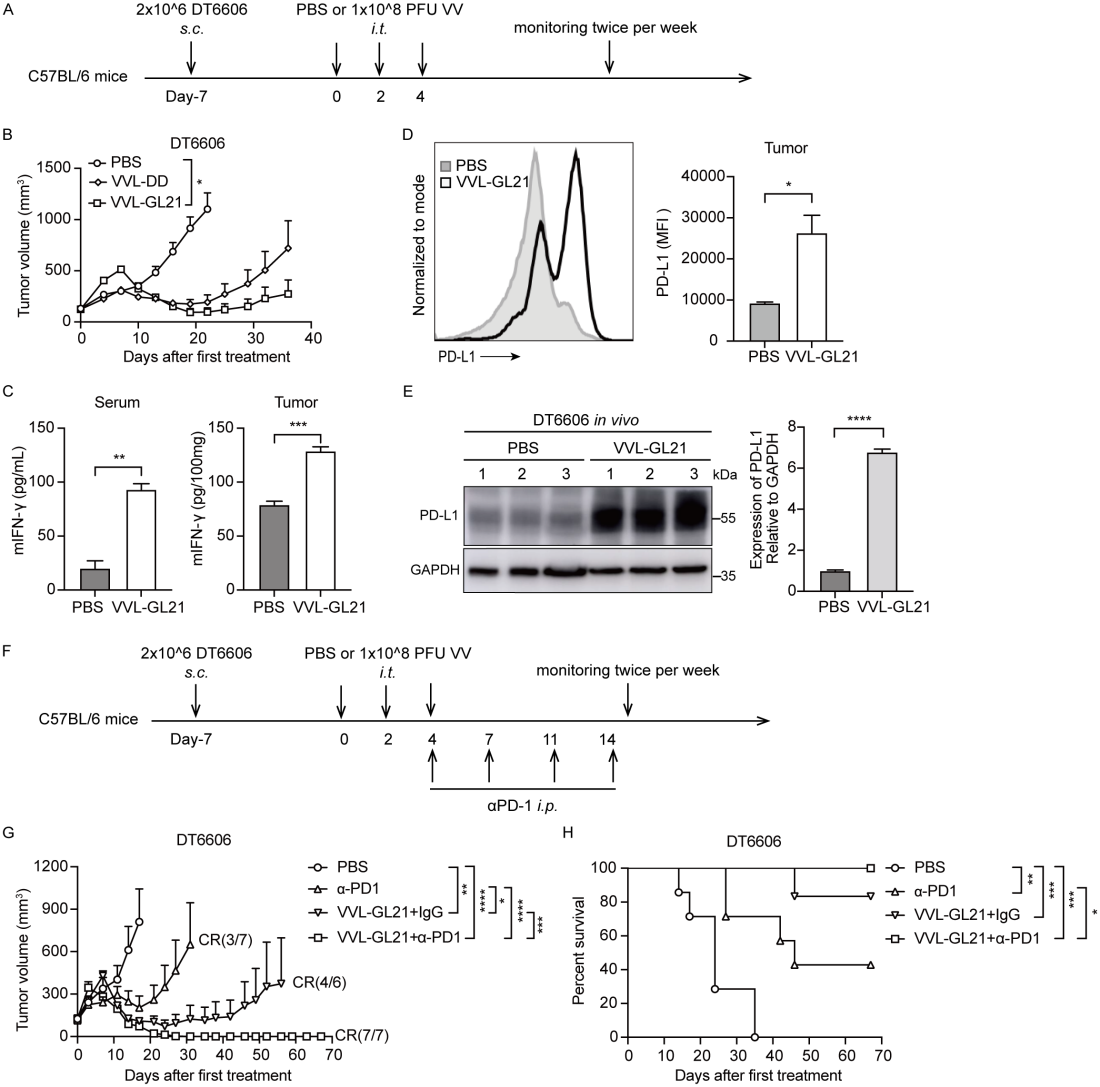
α-PD1 can augment the anti-tumor efficacy of intratumoral-delivered VVL-GL21 *in vivo*. **(A)** Schematic for the *in vivo* treatment protocol. Wild-type mice were subcutaneously engrafted with 2 × 10^6^ DT6606 cells. Seven days later (average tumor size 100 mm^3^), mice were treated with intratumoral injection of VVL-DD or VVL-GL21 (1 × 10^8^ PFU/injection) on days 0, 2, and 4; n = 7. **(B)** Tumor growth curve. **(C)** Tumors and blood were extracted from mice treated as **(A)**. Tumors were homogenized and sera were collected at 24 h after the last treatment. IFN-γ expression was detected using ELISA (n = 3). **(D, E)** A single cell suspension and total proteins of subcutaneous DT6606 tumors were prepared for FACS and western blot assay to assess PD-L1 expression, respectively (n = 3). **(F)** Schematic for the *in vivo* treatment protocol. DT6606 tumors were established subcutaneously and once palpable (120 mm^3^), were treated with intratumoral injection of VVL-GL21 (1 × 10^8^ PFU/injection) on days 0, 2, and 4. α-PD1 was administered via intraperitoneal injection on days 4, 7, 11, and 14 (200 μg/injection); n = 6/7. **(G)** Tumor growth curve. **(H)** Survival curve. Data are presented as the mean ± SEM. The data in **(B-E, G)** were analyzed using an unpaired Student’s t-test. The data in **(H)** were analyzed using Kaplan–Meier survival analysis with log rank (Mantel-Cox) tests. *p < 0.05, **p < 0.01, ***p < 0.001, ****p < 0.0001.

To increase the possibility of clinical translation and reduce medication costs, we investigated the potency of low-dose VVL-GL21 (**Figure 6A**). Although VVL-GL21 slowed down tumor growth, its ability to control tumor growth was significantly weakened (**Figure 6B**). Serum and tumor IFN-γ levels increased after VVL-GL21 treatment (**Figure 6C**). However, the IFN-γ level increase in the TME resulting from viral infection could upregulate the expression of PD-L1 in tumor cells (18). We found that DT6606, TB11381, DT4994, CMT64, and MC38 cells expressed low levels of PD-L1, which were significantly increased after IFN-γ treatment *in vitro* (**Supplementary Figures S6A, B**). FACS and western blot assays demonstrated that PD-L1 expression in the tumors significantly increased after VVL-GL21 treatment *in vivo* (**Figures 6D, E**, **Supplementary Figure S6C**). Further, FACS analysis showed the PD-L1 expression increased in tumor cells, myeloid cells and DCs (**Supplementary Figure S6D**).

Because of the lack of CD8+ T cells in the majority of tumor lesions, treatment with anti-PD1 or anti-PD-L1 antibodies shows clinical benefits only in a subset of patients (7). Our results showed that VVL-GL21 treatment significantly increased CD8+ T-cell infiltration and PD-L1 expression in tumors (**Figures 4A, B**, **6D, E**). We hypothesized that VVL-GL21 treatment could synergize with α-PD1 immunotherapy. We found that the combination of VVL-GL21 with α-PD1 cured all mice with no recurrence during the 105-day observation period (**Figures 6F–H**). FACS results indicated that VVL-GL21 and VVL-GL21 plus PD-1 antibody treatments expanded activated PD-1+Tim-3-CD8+ T cells and stem-like PD-1+TCF1+ precursor CD8+ T cells (**Supplementary Figure S6E**).

**Figure 7:**
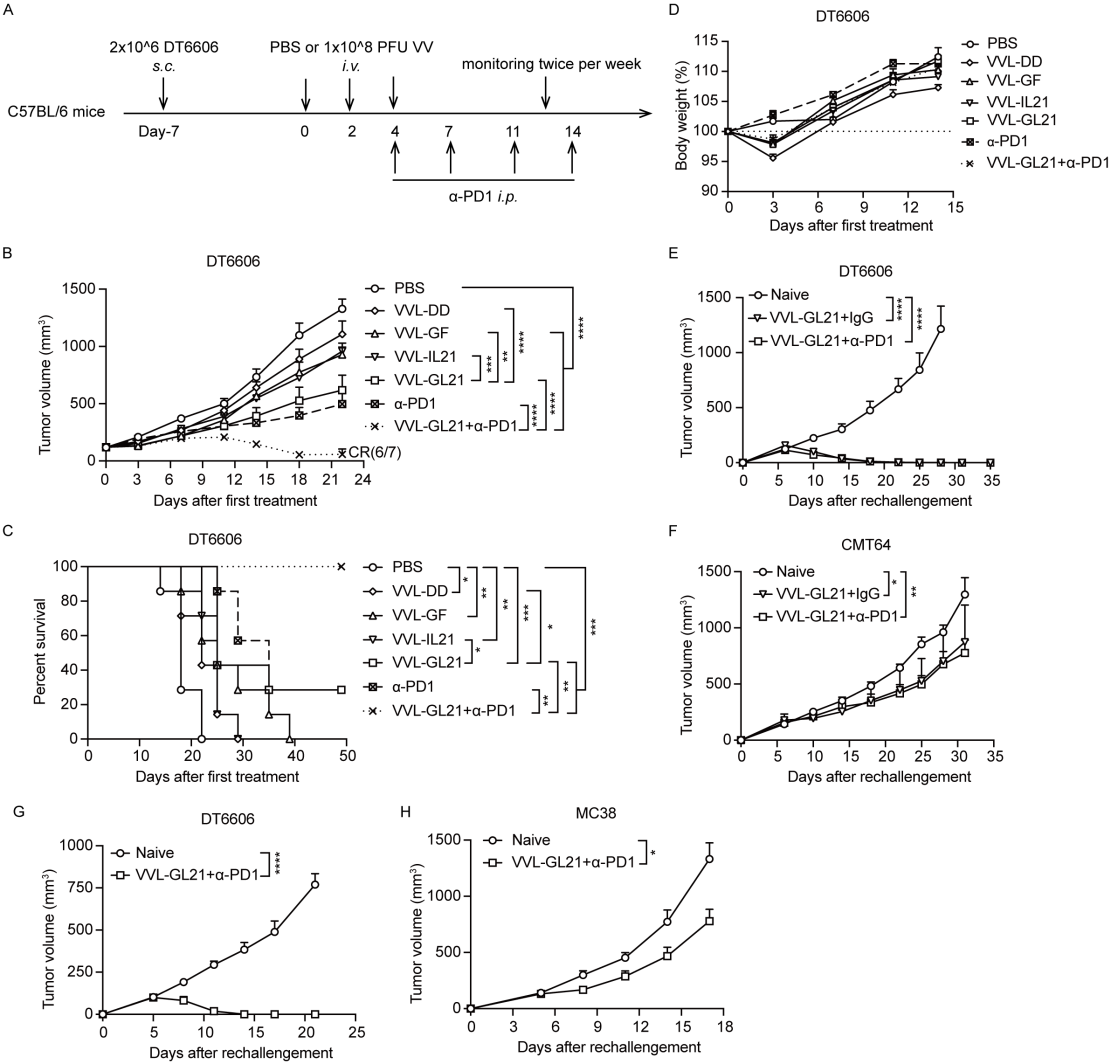
α-PD1 can augment the anti-tumor efficacy of intravenous-delivered VVL-GL21 *in vivo*. **(A)** Schematic for the *in vivo* treatment protocol. Wild-type mice were subcutaneously engrafted with 2 × 10^6^ DT6606 cells. Seven days later (average tumor size 100 mm^3^), mice were treated with intravenous injection of VVL-DD, VVL-GF, VVL-IL21 or VVL-GL21 (1 × 10^8^ PFU/injection) on days 0, 2, and 4. α-PD1 was administered by intraperitoneal injection on days 4, 7, 11, and 14 (200 μg/injection) (n = 7). **(B)** Tumor growth curve. **(C)** Survival curve. **(D)** Body weights. **(E, F)** Mice that had achieved tumor-free status after intratumoral treatment with VVL-GL21 or VVL-GL21+α-PD1 in the efficacy experiments were rechallenged 50 d later in the opposite flank with 4 × 10^6^ DT6606 and 5 × 10^6^ CMT64 cells. VVL-GL21 (n = 4); VVL-GL21+α-PD1 (n = 7). **(G, H)** Mice that had achieved tumor-free status after intravenous treatment with VVL-GL21+α-PD1 during the efficacy experiments were rechallenged 42 d later in the opposite flank with 4 × 10^6^ DT6606 or 5 × 10^5^ MC38 cells. VVL-GL21 +α-PD1 (n = 6). Data are presented as the mean ± SEM. Data in **(B)** were analyzed using two-way ANOVA with Tukey’s multiple comparison post-test. The data in **(E-H)** were analyzed using an unpaired Student’s t-test. The data in **(C)** were analyzed using Kaplan–Meier survival analysis with log rank (Mantel-Cox) tests. *p < 0.05, **p < 0.01, ***p < 0.001, ****p < 0.0001.

An advantage of VV is that it can be administered intravenously. To determine the anti-tumor efficacy of VVL-GL21 delivered by intravenous injection, established subcutaneous DT6606 tumors (120 mm^3^) were treated with viruses (1 × 10^8^ PFU/injection) and α-PD1 (**Figure 7A**). VVL-GL21 was significantly superior to other virus treatments in tumor suppression (**Figure 7B**). The combination of VVL-GL21 with α-PD1 therapy significantly inhibited tumor growth, with long-term survival in 6/7 mice treated (**Figures 7B, C**). There was no significant change in weight, indicating that intravenous injection of VVL-GL21 was safe (**Figure 7D**). These data support the suggestion that VVL-GL21 facilitates immune cell infiltration and therefore improved α-PD1-mediated anti-tumor efficacy.

### 3.8 VVL-GL21 promotes memory T-cell differentiation and prevents tumor recurrence

VVL-GL21 significantly promoted CD8+ Tem and CD8+ Tcm formation on days 7 and 14 after the first viral treatment (**Figure 4**). To assess whether these cells had robust anti-tumor effects and could prevent recurrence, 50 days after primary tumors were cleared by VVL-GL21 or VVL-GL21+α-PD1, mice were rechallenged with DT6606 (using twice as many cells as those originally inoculated) and CMT64 cells. Both VVL-GL21 and VVL-GL21 (intratumoral) +α-PD1 treated mice could reject DT6606 cells after the rechallenge and inhibited CMT64 tumors growth (**Figures 7E, F**). Similarly, tumor-free mice treated with VVL-GL21 (intravenous) +α-PD1 were rechallenged 42 days later with DT6606 and MC38 cells, and DT6606 tumors were rejected, and the growth of MC38 tumors was also inhibited (**Figures 7G, H**). These results were consistent with the ability of CMT64 and MC38 to induce IFN-γ release of splenocytes from VVL-GL21-treated mice (**Figure 5B**). These findings demonstrate that OV therapy eradicates primary tumors and induces long-term anti-tumor immunity to prevent tumor recurrence. *In vitro* and *in vivo* studies have shown that VVL-GL21 treatment has protective effects against other types of tumors, indicating the potential of VVL-GL21 as a therapeutic tumor vaccine.

## 4 Discussion

Limited therapeutic efficacy of VV has been observed in various clinical trials (12, 27). To overcome these limitations, a strategy is to optimize the anti-tumor potency of VV and reduce its virulence. VV encodes 16 proteins that intercept NF-κB activation, namely A46, A49, A52, B14, K7, F14, and N1L (28). Lack of individual inhibitors reduced VV virulence. Our group previously reported that deletion of the TK and N1L genes results in tumor selectivity for VV and further enhances the induction of anti-tumor immune responses through various mechanisms (10). The removal of the TK and A49 genes resulted in increased tumor selectivity *in vitro* and superior anti-tumor efficacy in a murine PaCa model.

Another strategy is to optimize the therapeutic gene expression based on the TME. Immune regulatory molecules (cytokines, chemokines, costimulatory factors, and ICI), metabolic enzymes, and enzymes that degrade the extracellular matrix (hyaluronidase) can be used to arm OVs (29–31). GM-CSF was expressed by VV to treat patients with cutaneous melanoma 25 years ago and was also inserted into oncolytic adenovirus or HSV to treat patients with cancers (32–34). GM-CSF promotes the maturation of DCs and boosts antigen presentation, which is the first signal of T-cell activation. Clinical trials of OVs expressing GM-CSF have demonstrated that GM-CSF is a safe, but limited effect therapeutic gene (15, 27, 35). These findings highlight the need to explore the use of therapeutic genes combined with GM-CSF to increase the anti-tumor efficacy of OVs. IL-21 has been extensively studied for its effects on the proliferation and functionality of T cells (20, 36, 37). Our group previously reported that VV armed with IL-21 exhibited effectiveness against tumors in murine PaCa, colorectal cancer, and glioma models (18, 38, 39). Here, we reported that VVL expressing GM-CSF and IL-21 and VVL-GL21 had outstanding effectiveness in combating aggressive PaCa tumor growth even in high tumor burden. The combination of GM-CSF and IL-21 in other OVs and cytokine therapies could improve treatment outcomes.

It is crucial for OVs to focus on developing strategies that can counteract the strongly immunosuppressive TME (40). We observed a significant increase in DCs and activated DCs within the TME and a notable increase in the total number of macrophages, particularly the M1 macrophage subset, in draining lymph nodes. IL-21 affects macrophages and can induce M2 to M1 polarization when delivered directly into tumors (39, 41). A correlation between M2 macrophages and poor prognosis has been observed in PaCa and other cancers (42). Furthermore, we found an increase in the number of T cells, particularly CD8+ Tems and Tcm cells, within the TME, draining lymph nodes, and spleen. These findings indicate that VVL-GL21 activates the systemic immune system and induces immune memory against tumors.

We observed that the splenocytes of mice bearing-DT6606 tumors treated with VVL-GL21 produced large amounts of IFN-γ against DT6606, CMT64, and MC38 from day 7 to day 14. *In vivo*, VVL-GL21 treatment prevented DT6606 tumor recurrence, and significantly inhibited CMT64 and MC38 tumor growth. These results indicate that the combination of GM-CSF and IL-21 generates a powerful anti-tumor response and immune memory in a short period, leading to a strong immune response that prevents tumor recurrence and significantly inhibits other types of tumors. This inhibitory effect may be due to the presence of cross-antigens and the formation of immune memory. This study provides a basis for developing VVL-GL21 as a therapeutic cancer vaccine.

T-VEC, when combined with anti-PD1 therapy, shows a significant response in patients with advanced melanoma (7). We found that intratumoral or intravenous injection of VVL-GL21 combined with α-PD1 cured murine subcutaneous PaCa tumors and induced strong immune memory. After VVL-GL21 treatment, a notable increase in PD-L1 levels in DT6606 tumor tissues was observed, potentially explaining the positive outcome of the combination therapy. Chen et al. reported that VV-expressing PD-L1 inhibitors and GM-CSF was capable of activating neoantigen-specific T cell responses through a likely synergistic action (43). In this study, we also noted that intravenous injection of VVL-GL21 had a superior therapeutic effect even without idelalisib (CAL-101) pre-treatment. Our group previously reported that transient PI3Kδ inhibition by CAL-101 enhanced the therapeutic effect of intravenous VV delivery by preventing macrophage uptake of the virus (44). Tumor growth may be controlled more effectively after intravenous injection of VVL-GL21 in combination with CAL-101 than after administration without CAL-101.

These findings suggest that the synergistic effect of GM-CSF and IL-21 significantly enhances systemic anti-tumor activity and that the VVL therapeutic platform can broaden the scope of ICI and cancer vaccine treatments.

## Data Availability

The original contributions presented in the study are included in the article/**Supplementary Material**. Further inquiries can be directed to the corresponding author.

## References

[1] Bray F Laversanne M Sung H Ferlay J Siegel RL Soerjomataram I. Global cancer statistics 2022: GLOBOCAN estimates of incidence and mortality worldwide for 36 cancers in 185 countries. CA Cancer J Clin. (2024) 74:229–63. doi: 10.3322/caac.21834 38572751

[2] Qi C Xie T Zhou J Wang X Gong J Zhang X. CT041 CAR T cell therapy for Claudin18.2-positive metastatic pancreatic cancer. J Hematol Oncol. (2023) 16:102. doi: 10.1186/s13045-023-01491-9 37689733 PMC10492318

[3] Tang HY Cao YZ Zhou YW Ma YS Jiang H Zhang H. The power and the promise of CAR-mediated cell immunotherapy for clinical application in pancreatic cancer. J Adv Res. (2024), S2090-1232(24)00027-4. doi: 10.1016/j.jare.2024.01.014 PMC11725162 38244773

[4] Hu ZI O’Reilly EM. Therapeutic developments in pancreatic cancer. Nat Rev Gastroenterol Hepatol. (2024) 21:7–24. doi: 10.1038/s41575-023-00840-w 37798442

[5] Shalhout SZ Miller DM Emerick KS Kaufman HL. Therapy with oncolytic viruses: progress and challenges. Nat Rev Clin Oncol. (2023) 20:160–77. doi: 10.1038/s41571-022-00719-w 36631681

[6] Johnson DB Puzanov I Kelley MC. Talimogene laherparepvec (T-VEC) for the treatment of advanced melanoma. Immunotherapy. (2015) 7:611–9. doi: 10.2217/imt.15.35 PMC4519012 26098919

[7] Ribas A Dummer R Puzanov I VanderWalde A Andtbacka RHI Michielin O. Oncolytic virotherapy promotes intratumoral T cell infiltration and improves anti-PD-1 immunotherapy. Cell. (2017) 170:1109–1119.e10. doi: 10.1016/j.cell.2017.08.027 28886381 PMC8034392

[8] Kim M Nitschké M Sennino B Murer P Schriver BJ Bell A. Amplification of oncolytic vaccinia virus widespread tumor cell killing by sunitinib through multiple mechanisms. Cancer Res. (2018) 78:922–37. doi: 10.1158/0008-5472.CAN-15-3308 PMC6501576 29259007

[9] Breitbach CJ Burke J Jonker D Stephenson J Haas AR Chow LQ. Intravenous delivery of a multi-mechanistic cancer-targeted oncolytic poxvirus in humans. Nature. (2011) 477:99–102. doi: 10.1038/nature10358 21886163

[10] Ahmed J Chard LS Yuan M Wang J Howells A Li Y. A new oncolytic Vacciniavirus augments antitumor immune responses to prevent tumor recurrence and metastasis after surgery. J Immunother Cancer. (2020) 8:e000415. doi: 10.1136/jitc-2019-000415 32217766 PMC7206973

[11] Hiley CT Chard LS Gangeswaran R Tysome JR Briat A Lemoine NR. Vascular endothelial growth factor A promotes vaccinia virus entry into host cells via activation of the Akt pathway. J Virol. (2013) 87:2781–90. doi: 10.1128/JVI.00854-12 PMC3571386 23269798

[12] Xu L Sun H Lemoine NR Xuan Y Wang P. Oncolytic vaccinia virus and cancer immunotherapy. Front Immunol. (2023) 14:1324744. doi: 10.3389/fimmu.2023.1324744 38283361 PMC10811104

[13] Toulmonde M Guegan JP Spalato-Ceruso M Peyraud F Kind M Vanhersecke L. Reshaping the tumor microenvironment of cold soft-tissue sarcomas with oncolytic viral therapy: a phase 2 trial of intratumoral JX-594 combined with avelumab and low-dose cyclophosphamide. Mol Cancer. (2024) 23:38. doi: 10.1186/s12943-024-01946-8 38378555 PMC10877825

[14] Toulmonde M Cousin S Kind M Guegan JP Bessede A Le Loarer F. Randomized phase 2 trial of intravenous oncolytic virus JX-594 combined with low-dose cyclophosphamide in patients with advanced soft-tissue sarcoma. J Hematol Oncol. (2022) 15:149. doi: 10.1186/s13045-022-01370-9 36271420 PMC9585864

[15] Abou-Alfa GK Galle PR Chao Y Erinjeri J Heo J Borad MJ. PHOCUS: A phase 3, randomized, open-label study of sequential treatment with pexa-vec (JX-594) and sorafenib in patients with advanced hepatocellular carcinoma. Liver Cancer. (2024) 13:248–64. doi: 10.1159/000533650 PMC11095598 38756145

[16] Hughes J Wang P Alusi G Shi H Chu Y Wang J. Lister strain vaccinia virus with thymidine kinase gene deletion is a tractable platform for development of a new generation of oncolytic virus. Gene Ther. (2015) 22:476–84. doi: 10.1038/gt.2015.13 25876464

[17] Neidel S Ren H Torres AA Smith GL. NF-kappaB activation is a turn on for vaccinia virus phosphoprotein A49 to turn off NF-kappaB activation. Proc Natl Acad Sci USA. (2019) 116:5699–704. doi: 10.1073/pnas.1813504116 PMC6431142 30819886

[18] Sun Y Zhang Z Zhang C Zhang N Wang P Chu Y. An effective therapeutic regime for treatment of glioma using oncolytic vaccinia virus expressing IL-21 in combination with immune checkpoint inhibition. Mol Ther Oncolytics. (2022) 26:105–19. doi: 10.1016/j.omto.2022.05.008 PMC9233193 35795092

[19] Chen T Ding X Liao Q Gao N Chen Y Zhao C. IL-21 arming potentiates the anti-tumor activity of an oncolytic vaccinia virus in monotherapy and combination therapy. J Immunother Cancer. (2021) 9:e001647. doi: 10.1136/jitc-2020-001647 33504576 PMC7843316

[20] Schmidt H Brown J Mouritzen U Selby P Fode K Svane IM. Safety and clinical effect of subcutaneous human interleukin-21 in patients with metastatic melanoma or renal cell carcinoma: a phase I trial. Clin Cancer Res. (2010) 16:5312–9. doi: 10.1158/1078-0432.CCR-10-1809 20959407

[21] Yuan M Zhang W Wang J Al Yaghchi C Ahmed J Chard L. Efficiently editing the vaccinia virus genome by using the CRISPR-Cas9 system. J Virol. (2015) 89:5176–9. doi: 10.1128/JVI.00339-15 PMC4403460 25741005

[22] Yuan M Gao X Chard LS Ali Z Ahmed J Li Y. A marker-free system for highly efficient construction of vaccinia virus vectors using CRISPR Cas9. Mol Ther Methods Clin Dev. (2015) 2:15035. doi: 10.1038/mtm.2015.35 26417609 PMC4571730

[23] Mali P Yang L Esvelt KM Aach J Guell M DiCarlo JE. RNA-guided human genome engineering via Cas9. Science. (2013) 339:823–6. doi: 10.1126/science.1232033 PMC3712628 23287722

[24] Gao X Tsang JC Gaba F Wu D Lu L Liu P. Comparison of TALE designer transcription factors and the CRISPR/dCas9 in regulation of gene expression by targeting enhancers. Nucleic Acids Res. (2014) 42:e155. doi: 10.1093/nar/gku836 25223790 PMC4227760

[25] Chard LS Maniati E Wang P Zhang Z Gao D Wang J. A vaccinia virus armed with interleukin-10 is a promising therapeutic agent for treatment of murine pancreatic cancer. Clin Cancer Res. (2015) 21:405–16. doi: 10.1158/1078-0432.CCR-14-0464 25416195

[26] Yuan M Wang P Chard LS Lemoine NR Wang Y. A simple and efficient approach to construct mutant vaccinia virus vectors. J Vis Exp. (2016) 116:54171. doi: 10.3791/54171-v PMC5226142 27842362

[27] Kaufman HL Kohlhapp FJ Zloza A. Oncolytic viruses: a new class of immunotherapy drugs. Nat Rev Drug Discovery. (2015) 14:642–62. doi: 10.1038/nrd4663 PMC7097180 26323545

[28] Albarnaz JD Ren H Torres AA Shmeleva EV Melo CA Bannister AJ. Molecular mimicry of NF-kappaB by vaccinia virus protein enables selective inhibition of antiviral responses. Nat Microbiol. (2022) 7:154–68. doi: 10.1038/s41564-021-01004-9 PMC7614822 34949827

[29] Kurokawa C Agrawal S Mitra A Galvani E Burke S Varshine A. Mediation of antitumor activity by AZD4820 oncolytic vaccinia virus encoding IL-12. Mol Ther Oncol. (2024) 32:200758. doi: 10.1016/j.omton.2023.200758 38596304 PMC10869731

[30] Wei W Tian L Zheng X Zhong L Chen Y Dong H. Expression of GPX4 by oncolytic vaccinia virus can significantly enhance CD8(+)T cell function and its impact against pancreatic ductal adenocarcinoma. Oncoimmunology. (2024) 13:2322173. doi: 10.1080/2162402X.2024.2322173 38419758 PMC10900272

[31] Wang S Li Y Xu C Dong J Wei J. An oncolytic vaccinia virus encoding hyaluronidase reshapes the extracellular matrix to enhance cancer chemotherapy and immunotherapy. J Immunother Cancer. (2024) 12:e008431. doi: 10.1136/jitc-2023-008431 38458640 PMC10921532

[32] Cerullo V Pesonen S Diaconu I Escutenaire S Arstila PT Ugolini M. Oncolytic adenovirus coding for granulocyte macrophage colony-stimulating factor induces antitumoral immunity in cancer patients. Cancer Res. (2010) 70:4297–309. doi: 10.1158/0008-5472.CAN-09-3567 20484030

[33] Mastrangelo MJ. Intratumoral recombinant GM-CSF-encoding virus as gene therapy in patients with cutaneous melanoma. Cancer Gene Ther. (1999) 6:409–22. doi: 10.1038/sj.cgt.7700066 10505851

[34] Hu JC Coffin RS Davis CJ Graham NJ Groves N Guest PJ. A phase I study of OncoVEXGM-CSF, a second-generation oncolytic herpes simplex virus expressing granulocyte macrophage colony-stimulating factor. Clin Cancer Res. (2006) 12:6737–47. doi: 10.1158/1078-0432.CCR-06-0759 17121894

[35] Andtbacka RH Kaufman HL Collichio F Amatruda T Senzer N Chesney J. Talimogene laherparepvec improves durable response rate in patients with advanced melanoma. J Clin Oncol. (2015) 33:2780–8. doi: 10.1200/JCO.2014.58.3377 26014293

[36] Thompson JA Curti BD Redman BG Bhatia S Weber JS Agarwala SS. Phase I study of recombinant interleukin-21 in patients with metastatic melanoma and renal cell carcinoma. J Clin Oncol. (2008) 26:2034–9. doi: 10.1200/JCO.2007.14.5193 18347008

[37] Isvoranu G Chiritoiu-Butnaru M. Therapeutic potential of interleukin-21 in cancer. Front Immunol. (2024) 15:1369743. doi: 10.3389/fimmu.2024.1369743 38638431 PMC11024325

[38] Wang N Wang J Zhang Z Cao H Yan W Chu Y. A novel vaccinia virus enhances anti-tumor efficacy and promotes a long-term anti-tumor response in a murine model of colorectal cancer. Mol Ther Oncolytics. (2021) 20:71–81. doi: 10.1016/j.omto.2020.11.002 33575472 PMC7851495

[39] Marelli G Chard Dunmall LS Yuan M Di Gioia C Miao J Cheng Z. A systemically deliverable Vaccinia virus with increased capacity for intertumoral and intratumoral spread effectively treats pancreatic cancer. J Immunother Cancer. (2021) 9. doi: 10.1136/jitc-2020-001624 PMC7839893 33500259

[40] DePeaux K Delgoffe GM. Integrating innate and adaptive immunity in oncolytic virus therapy. Trends Cancer. (2024) 10:135–46. doi: 10.1016/j.trecan.2023.09.012 PMC10922271 37880008

[41] Xu M Liu M Du X Li S Li H Li X. Intratumoral delivery of IL-21 overcomes anti-her2/neu resistance through shifting tumor-associated macrophages from M2 to M1 phenotype. J Immunol. (2015) 194:4997–5006. doi: 10.4049/jimmunol.1402603 25876763

[42] Kurahara H Shinchi H Mataki Y Maemura K Noma H Kubo F. Significance of M2-polarized tumor-associated macrophage in pancreatic cancer. J Surg Res. (2011) 167:e211–9. doi: 10.1016/j.jss.2009.05.026 19765725

[43] Wang G Kang X Chen KS Jehng T Jones L Chen J. An engineered oncolytic virus expressing PD-L1 inhibitors activates tumor neoantigen-specific T cell responses. Nat Commun. (2020) 11:1395. doi: 10.1038/s41467-020-15229-5 32170083 PMC7070065

[44] Ferguson MS Chard Dunmall LS Gangeswaran R Marelli G Tysome JR Burns E. Transient inhibition of PI3Kdelta enhances the therapeutic effect of intravenous delivery of oncolytic vaccinia virus. Mol Ther. (2020) 28:1263–75. doi: 10.1016/j.ymthe.2020.02.017 PMC7210704 32145202

