# Supplementary material for: GM-CSF and IL-21-armed oncolytic vaccinia virus significantly enhances anti-tumor activity and synergizes with anti-PD1 immunotherapy in pancreatic cancer
Source: Front Immunol. 2025 Jan 3;15:1506632. doi: 10.3389/fimmu.2024.1506632 (PMC11739091; doi:10.3389/fimmu.2024.1506632)

**Supplemental Figure S1-6**

**Supplemental Figure S1.** **Schematic detailing the construction of mutant viruses.** TK and A49 regions were replaced by RFP expression cassette, and GM-CSF expression cassette or IL-21 expression cassette as indication. The exogenous genes were under control of the endogenous H5 promoter to drive high level, stable gene expression.

**Supplemental Figure S2. VVL-GL21 improves in vivo anti-tumor efficacy in murine pancreatic model.** (**A**) Schematic for the in vivo treatment protocol. TB11381 tumors were established subcutaneously and once palpable (160 mm^3^), were treated with intratumoral injection of VVL-DD, VVL-GF, VVL-IL21 or VVL-GL21 (1 × 10^8^ PFU/injection) on days 0, 2, and 4 (n = 7). (**B**) Tumor growth curve. (**C**) Survival curve. (**D**) Body weight measurements. Data are presented as the mean ± SEM. The data in (B) were analyzed using a two-way ANOVA with Tukey’s multiple comparison post-test. The data in (C) were analyzed using Kaplan–Meier survival analysis with log rank (Mantel-Cox) tests. *p < 0.05, **p < 0.01, ***p < 0.001, ****p < 0.0001.

**Supplemental Figure S3. FACS gating strategy for adaptive and innate immune populations. (A) T and NKT cells, (B) DCs and macrophages.**

**Supplemental Figure S4. VVL-GL21 therapy changes percentage of immune cells in tumor, spleen, and draining lymph node.** (**A**) Representative images of immunohistochemical (IHC) staining for CD8+ T cells in DT6606 subcutaneous tumors collected on days 7 and 14, n = 3. Scale bars: 50 μm. (**B**) CD8+ T cells density was calculated in five high-power fields from each tumor section (× 20). (**C-D**) Spleens and draining lymph nodes were extracted from mice on day 14 after the first treatment. FACS were performed to analyze the immune cells using single cell suspensions prepared from these tissues (n = 3). (**E**) Representative images of hematoxylin-eosin (HE) staining for lung, liver, kidney and heart. Scale bars: 50 μm. (F) MC38 tumors were established and treated on day 0 with 2 × 10^8^ PFU of VVL-DD, VVL-GF, VVL-IL21, or VVL-GL21. Tumors were collected from mice on day 7 post-treatment. FACS was performed to analyze immune cell composition using single-cell suspensions prepared from these tissues (n=3). Data are presented as the mean ± SEM. Data were analyzed using an unpaired Student’s t-test. *p < 0.05, **p < 0.01.

**Supplemental Figure S5. FACS data support the efficacy of specific antibody-mediated depletion of immune cell subsets.**

**Supplemental Figure S6. The PD-L1 expression in different cells and exhaustion status of CD8+ T cells. (A–B**) The indicated cells were seeded in six-well plates containing the appropriate units/ml (U/ml) IFN-γ. After 60 h, cells were collected and processed for FACS analysis to assess PD-L1 expression. The mean fluorescence intensity of cells stained with PE conjugated anti-body recognizing PD-L1 is shown; n = 3. (C) The FACS gating strategy for PD-L1 expression in tumors. (D) The PD-L1 expression in tumor cells, myeloid cells and DC1 (n = 3). (E) The Tim-3+PD-1+ T cell, Tim-3-PD-1+ T cell and PD-1+ TCF1+ T cell populations in TME after VVL-GL21 and VVL-GL21 plus PD-1 antibody treatments (n = 3). Data are presented as the mean ± SEM. Data were analyzed using an unpaired Student’s t-test. *p < 0.05, **p < 0.01, ***p < 0.001, ****p < 0.0001.


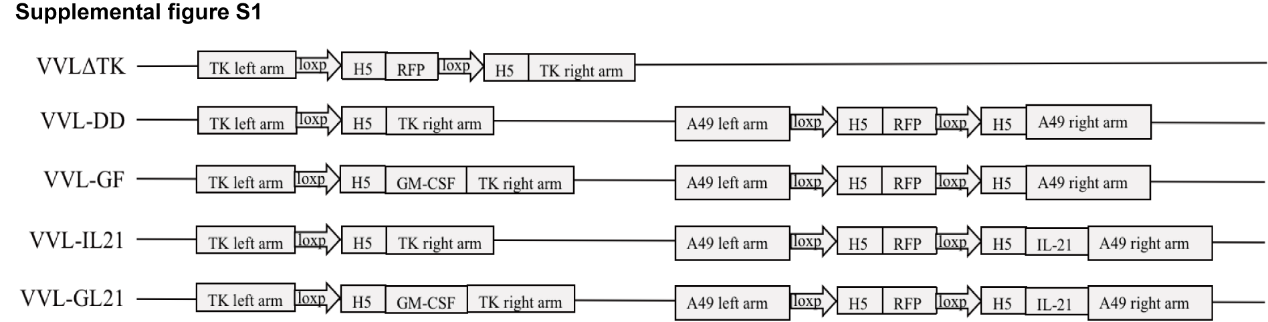


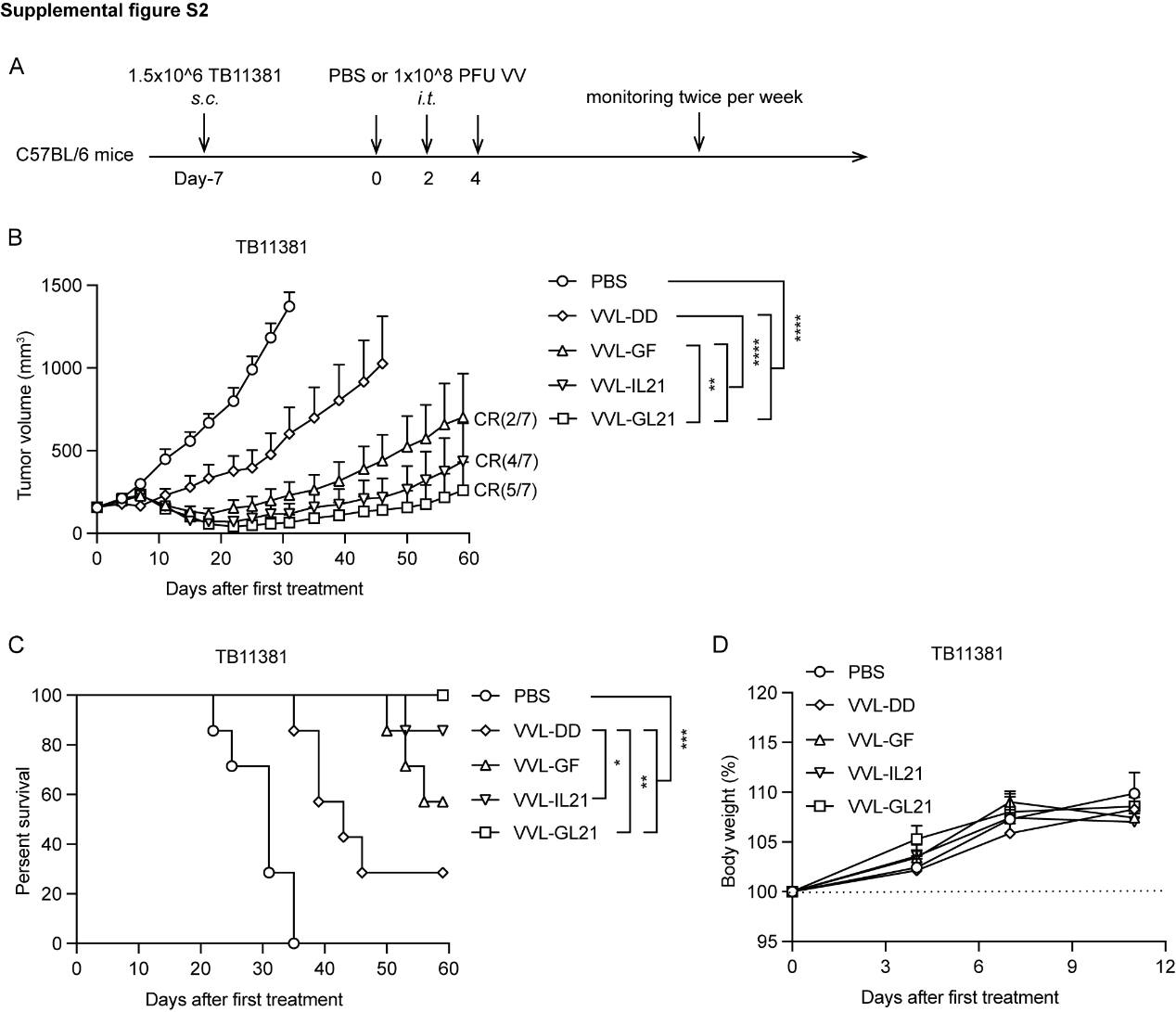


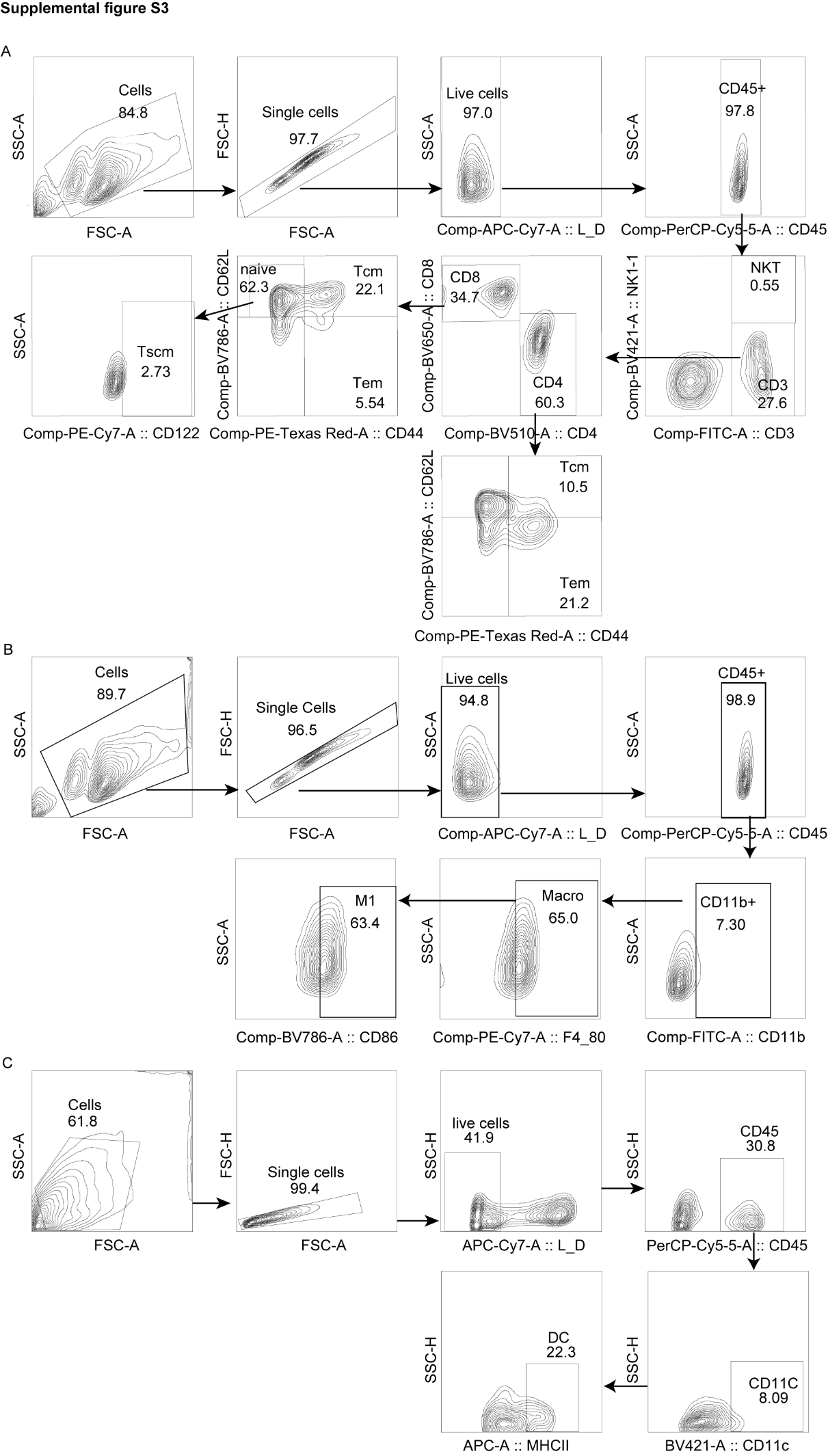


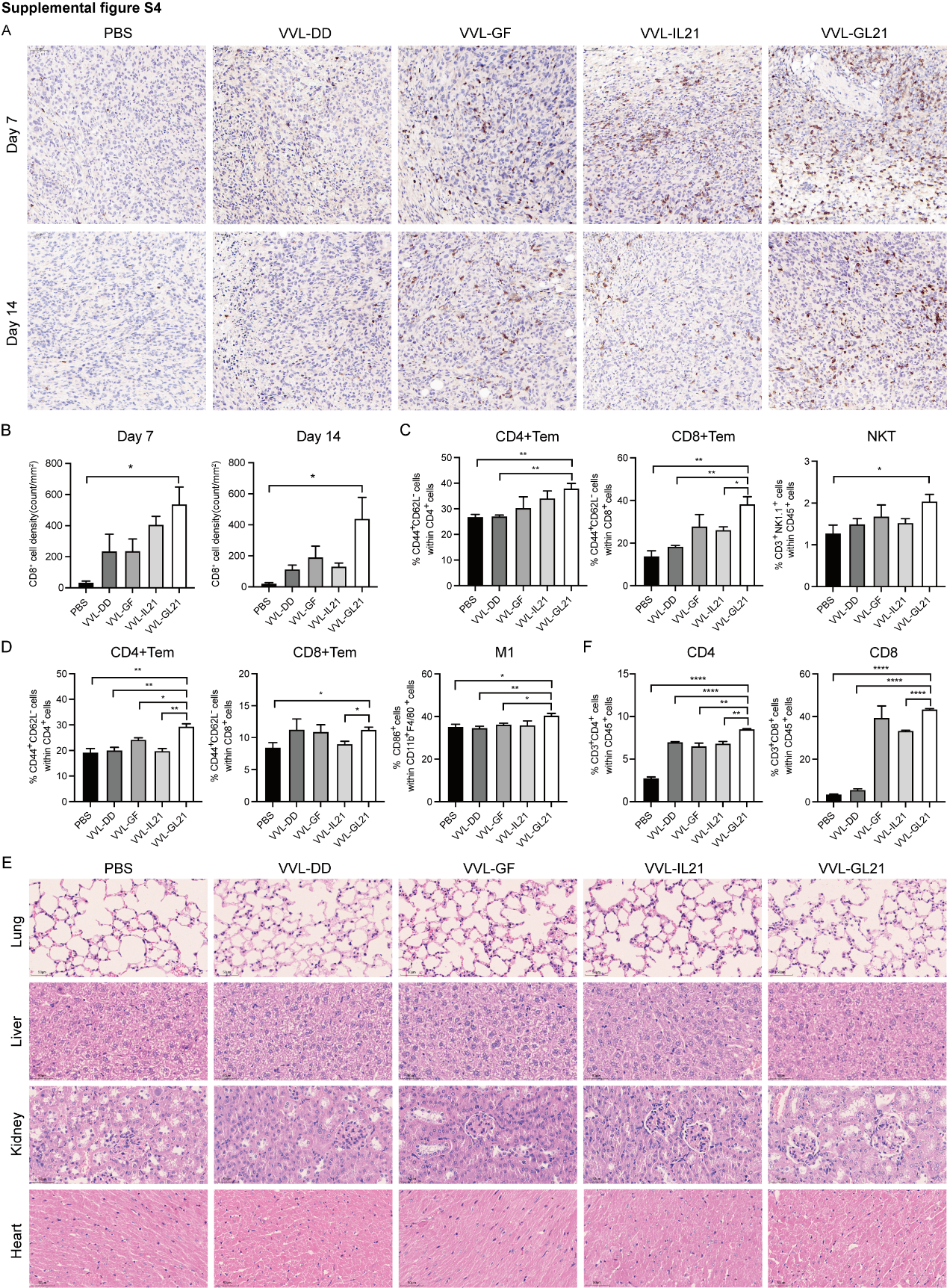


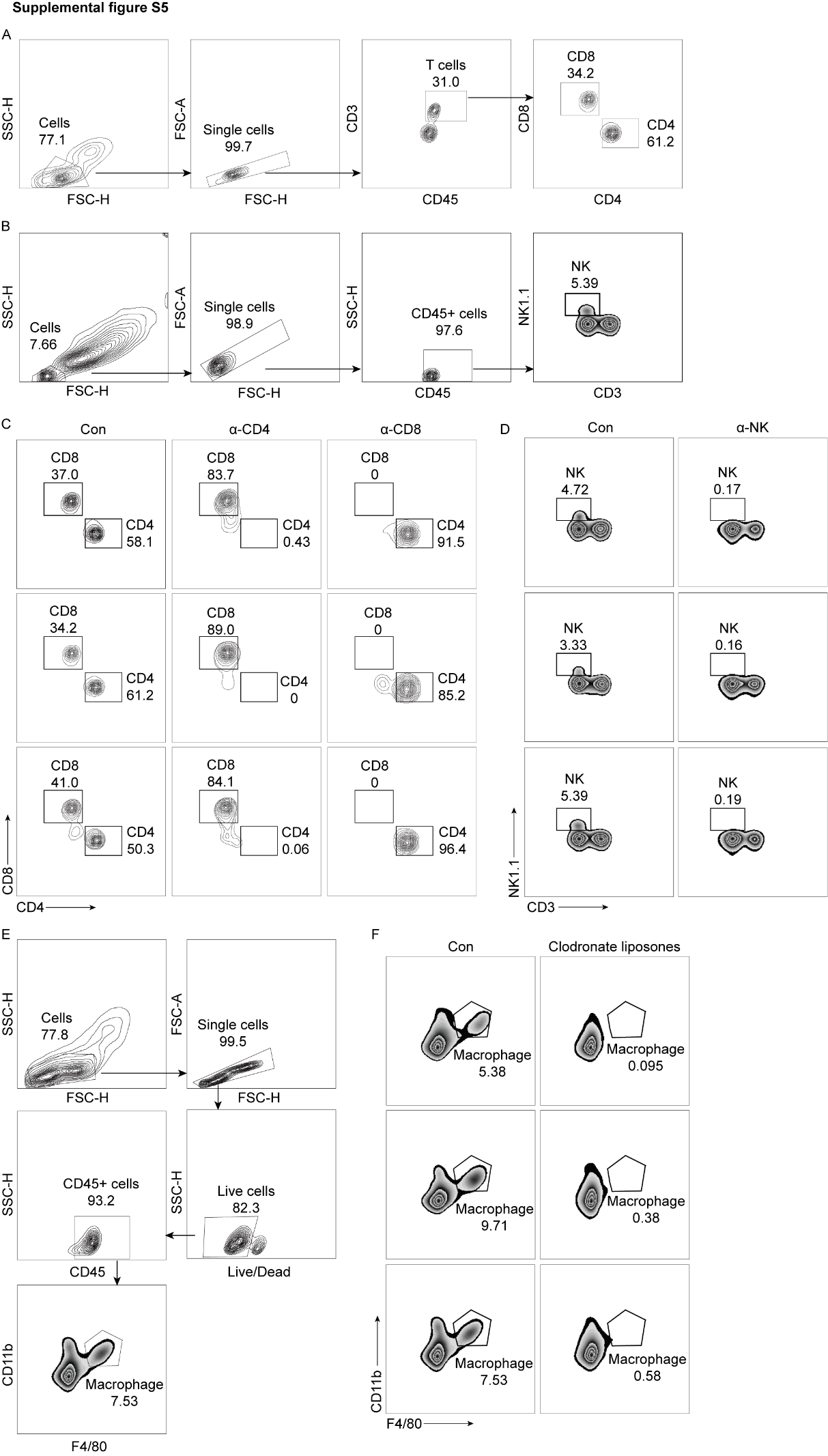


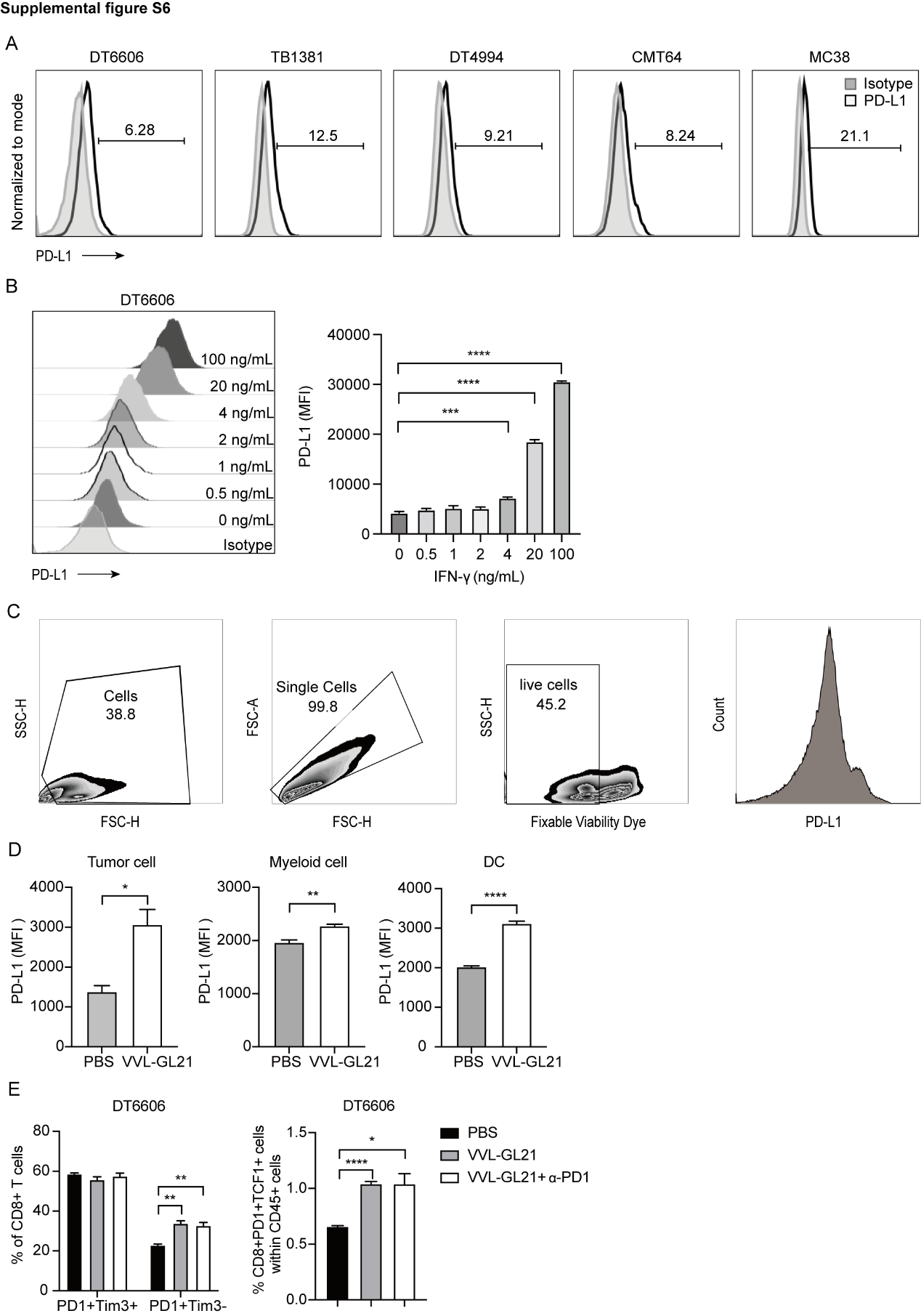

Supplement: Supplementary file 1 [file DataSheet1.docx]
